# Neuroprotective role of pyrroloquinoline quinone in folate deficiency-induced blood-brain barrier disruption

**DOI:** 10.1186/s12987-025-00689-y

**Published:** 2025-07-22

**Authors:** Sara Aboulhassane, Vishal Sangha, Md. Tozammel Hoque, Reina Bendayan

**Affiliations:** Department of Pharmaceutical Sciences, Leslie Dan Faculty of Pharmacy, University of Toronto, Toronto, ON Canada

**Keywords:** Pyrroloquinoline quinone, Blood-brain barrier, Cerebral folate deficiency, Neuroinflammation, Oxidative stress, BBB permeability, Folate transporters, Tight-Junction proteins

## Abstract

Healthy neurodevelopment requires adequate folates (vitamin B9), which are critical for key biosynthetic and homeostatic processes in the central nervous system (CNS). In the brain, folate transport is mediated by three major pathways: folate receptor alpha (FRα), proton-coupled folate transporter (PCFT), and reduced folate carrier (RFC). Folate uptake primarily occurs at the blood-cerebrospinal fluid barrier (BCSFB) by concerted actions of FRα and PCFT. Alterations in this transport pathway can result in cerebral folate deficiency (CFD), a rare but devastating pediatric condition associated with neuroinflammation and oxidative stress. Recent findings highlight the blood-brain barrier (BBB) as an alternative route for folate delivery, particularly through RFC upregulation. We hypothesized that pyrroloquinoline quinone (PQQ), an activator of nuclear respiratory factor 1 (NRF-1) and PGC-1α, key regulators of mitochondrial biogenesis, could enhance RFC expression at the BBB and mitigate CFD-induced damage. Using *in vitro* and *in vivo* models of folate deficiency, we investigated its impact on BBB integrity, inflammation, oxidative stress, mitochondrial dysfunction, and assessed PQQ’s ability to reverse these effects. Human brain microvessel endothelial cells (hCMEC/D3) cultured in control folate-sufficient (FS) or folate-deficient (FD) medium were treated with PQQ (1 or 5 µM) or vehicle control for 24 h. Wildtype (C57BL6/N) mice received FD (0 mg/kg folate), or FS (2 mg/kg folate) diet and underwent a 10-day (20 mg/kg/day, i.p) PQQ treatment. Following treatment, hCMEC/D3 cells and isolated mouse brain capillaries were analyzed using qPCR, ELISA, and immunoblotting to assess gene and protein expression of tight junction proteins, inflammatory and oxidative stress markers, mitochondrial transcription factors, and folate transporters. BBB permeability was evaluated *in vivo* using the sodium fluorescein (NaFl) assay. FD significantly increased the gene and/or protein expression of inflammatory cytokines/chemokines, endothelial adhesion molecules and oxidative stress markers, while tight junction proteins were significantly downregulated both in vitro and in vivo. The NaFl assay confirmed increased BBB permeability in FD mice. PQQ treatment effectively reversed these changes by upregulating RFC and PCFT expression, restoring BBB permeability, mitigating inflammatory and oxidative stress responses and improving mitochondrial biogenesis via PGC-1α/NRF-1 signaling. These results highlight the impact of brain FD on BBB integrity, potentially contributing to neurological deficits seen in CFD disorders with PQQ providing a promising therapeutic strategy.

**Supplementary Information:**

The online version contains supplementary material available at 10.1186/s12987-025-00689-y.

## Introduction

Folates (vitamin B9) are essential micronutrients required for DNA synthesis and repair, amino acid production, and other aspects of cellular metabolism and regulation that support key biosynthetic and homeostatic processes in the CNS and systemic tissues [1]. Adequate folate levels are particularly important during neurodevelopment, as folate deficiency (FD) is a significant risk factor for neural tube defects (NTDs), congenital heart defects, neurodevelopmental and neurodegenerative disorders [2–5]. Due to its high hydrophilicity and bivalent anionic nature at physiological pH, folates cannot diffuse passively across cell membranes, and therefore require specialized transport mechanisms [6, 7]. In mammals, folate transport across biological membranes is mediated by three major pathways, each with specific affinity and function i.e., folate receptors (FRα and FRβ; FOLR1-2), the proton-coupled folate transporter (PCFT; SLC46A1), and the reduced folate carrier (RFC; SLC19A1) [8–10]. Cerebral folate transport predominantly occurs at the choroid plexus (CP) epithelium of the blood-cerebrospinal fluid barrier (BCSFB), by the combined action of FRα and PCFT [11–13]. Disruptions in folate transport at this interface, particularly caused by mutations in the FOLR1 gene or the presence of FRα autoantibodies, lead to reduced folate levels in the cerebrospinal fluid (CSF), a key factor in neurodevelopmental disorders such as cerebral folate deficiency (CFD) [14–16]. Furthermore, low CSF folate levels and a high prevalence of FRα autoantibodies have been documented in neurological conditions, including autism spectrum disorder (ASD) [17, 18].

While the BCSFB has been recognized as the primary site for CNS folate transport, recent findings from our group have identified the blood-brain barrier (BBB) as an additional route in the context of CFD, highlighting its potential as a therapeutic target for restoring brain folate levels when transport through the BCSFB is impaired [19–22]. We have previously demonstrated that activation of vitamin D receptor (VDR) by its natural ligand, calcitriol, in FRα knockout mice can enhance RFC expression and function at the BBB, resulting in a significant increase in folate uptake in the brain [20]. In addition, we have recently identified that the transcription factor nuclear respiratory factor 1 (NRF-1), which regulates mitochondrial function, is another key regulator of RFC functional expression [21]. In particular, activation of NRF-1 by pyrroloquinoline quinone (PQQ), a naturally derived enzyme cofactor, enhanced RFC function in both *in vitro* and *in vivo* models of the BBB [21]. These findings suggest that functional upregulation of RFC at the BBB could serve as a potential strategy to improve cerebral folate delivery in conditions where folate transport at the BCSFB is impaired [19–21]. However, while studies on RFC upregulation at the BBB have shown promise for enhancing brain folate delivery, the impact of FD on BBB structural and functional integrity remains unclear.

The BBB is the largest interface for blood–brain exchange of nutrients and a highly specialized, selectively permeable barrier that regulates molecular transport between the peripheral circulation and the CNS [23, 24]. Structurally, this barrier is formed by a monolayer of non-fenestrated brain microvascular endothelial cells, supported by pericytes and astrocyte end-feet [25–27]. The integrity of the BBB is maintained by intercellular junctions, including tight junctions (TJs) and adherens junctions (AJs), which collectively restrict paracellular diffusion, thereby limiting the transport of endogenous and exogenous substances [28]. TJs are primarily composed of transmembrane proteins occludin (OCLN) and claudin-5 (CLDN-5), and the accessory protein zonula occludens − 1 (ZO-1/TJP1) which anchors TJ proteins complex to the actin cytoskeleton, stabilizing transmembrane proteins and maintaining structural integrity of endothelial cells [29–31]. Additionally, AJs are composed of cadherins and catenins, which further contribute to BBB stability by reinforcing cell-cell adhesion, regulating intracellular signaling, and interacting with TJs to modulate barrier permeability [28, 32]. Furthermore, the BBB features specialized transport systems, including ATP-binding cassette (ABC) membrane-associated efflux transporters, such as P-glycoprotein (P-gp) and Breast Cancer Resistance Protein (BCRP), which actively prevent potentially harmful substances from entering the brain parenchyma from the systemic circulation, and solute carrier (SLC) transporters, like the glucose transporter 1 (Glut-1), which facilitate the uptake of essential nutrients such as glucose [33, 34]. Together, these structural and transport mechanisms preserve BBB integrity, ensuring neural protection and maintaining CNS homeostasis. It is well established that dysfunction of the BBB is associated with several neurological disorders, including multiple sclerosis, Parkinson’s and Alzheimer’s disease [35, 36].

Evidence suggests that elevated homocysteine levels, a metabolic consequence of FD, downregulate the expression of TJ proteins and impair BBB function [37]. In addition, due to the essential role of folates in various biosynthetic processes, FD has also been associated with CNS inflammation, oxidative stress, and mitochondrial dysfunction, including increased mitochondrial DNA (mtDNA) deletions and impaired activity of mitochondrial enzymes [40–42]. Neurological disorders associated with brain FD, such as CFD, ASD and mitochondrial diseases, often present with these pathophysiological changes [14, 41, 42]. Recent studies from our group showed that FD induces brain inflammation and oxidative stress both *in vitro*, in primary cultures of mixed glial cells, and *in vivo*, in wild-type mice fed a FD diet [43]. Additionally, a reduction in mtDNA content was observed in FD-treated mixed glial cells [43]. While the BBB is recognized as a key site for folate delivery to the CNS from the peripheral circulation, the direct impact of FD on BBB structure and function, to the best of our knowledge, has not been thoroughly investigated.

In addition to upregulating RFC expression at the BBB by indirectly activating the NRF-1/Peroxisome proliferator-activated receptor gamma coactivator 1-alpha (PGC-1α) signaling pathway, PQQ has been recognized for its neuroprotective effects [43]. PQQ has gained attention for its anti-inflammatory and antioxidant properties, along with its ability to improve mitochondrial function [44–46]. Furthermore, it has also been shown to upregulate TJ proteins in porcine intestinal epithelial cells, suggesting a potential role in enhancing barrier integrity [47]. In the CNS, PQQ has demonstrated neuroprotective effects in several *in vitro *and *in vivo *models of brain injury and disease, making it a promising therapeutic candidate for the treatment of neurological disorders [48–51].

The aim of the present study was to investigate the effect of FD on BBB integrity by assessing alterations in mitochondrial function, inflammation, oxidative stress, and folate transporter expression. Additionally, we evaluated the neuroprotective role of PQQ in reversing these effects and inducing changes in BBB integrity in FD conditions through the activation of key signaling pathways such as NRF-1/PGC-1α. This study provides novel insight into how BBB disruption may contribute to the neurological deficits observed in CFD and elucidates PQQ’s potential as a neuroprotective agent for conditions associated with FD and BBB dysfunction.

## Materials & methods

### Materials

All cell culture reagents used for *in vitro* experiments were purchased from Invitrogen (Carlsbad, CA, USA) unless indicated otherwise. PQQ was obtained from Cayman Chemical (Ann Arbor, MI, USA). Real time quantitative polymerase chain reaction (qPCR) reagents, including reverse transcription cDNA kits and qPCR TaqMan primers, were purchased from Life technologies (Carlsbad, CA, USA). For western blot experiments, primary rabbit polyclonal anti-SLC19A1 (AV44167), and anti-SLC46A1 (PCFT, SAB2108339) antibodies were purchased from Sigma-Aldrich. Primary rabbit polyclonal anti-ZO-1 (TJP1) antibody (AB96587) was obtained from Abcam (Cambridge, MA, USA). Primary Mouse monoclonal anti-β-actin antibody (sc-517582) was obtained from Santa Cruz Biotechnology. Primary rabbit polyclonal anti-OCLN (Occludin, 71-1500), and anti-CLDN5 (Claudin-5, 34-1600) antibodies as well as anti-rabbit Alexa Fluor 594 and anti-mouse Alexa Fluor 488–conjugated secondary antibodies were purchased from Invitrogen (Carlsbad, CA, USA). For ELISA experiments, the LEGEND MAX™ Human IL-6 ELISA Kit (430507), LEGEND MAX™ Human IL-8 ELISA Kit (41507), LEGEND MAX™ Human CCL2 (MCP-1) (438807) and LEGEND MAX™ Human CXCL10 (IP-10) (433607) were purchased from BioLegend (San Diego, CA, USA). Ficoll (Polysucrose 400) was obtained from BioShop (Burlington, ON, CA) and PluriStrainer 30 μm was purchased from PluriSelect Life Science (EI Cajon, CA, USA). 3-(4,5-dimethylthiazol-2-yl)-2,5-diphenyltetrazolium bromide (MTT) was purchased from Sigma-Aldrich (Oakville, ON, Canada).

### Cell culture

*In vitro* studies were performed in human brain microvessel endothelial cells (hCMEC/D3) kindly provided by Dr. Pierre-Olivier Couraud (Institut Cochin, Departement Biologie Cellulaire and INSERM, Paris, France). Briefly, cells were grown on rat tail collagen-coated flasks in Endothelial Cell Basal Medium-2 (Wisent Inc, Montreal, QC, Canada) supplemented with vascular endothelial growth factor, insulin-like growth factor 1, epidermal growth factor, fibroblast growth factors, hydrocortisone, ascorbate, GA-1000, heparin and 2.5% of fetal bovine serum. For FD studies, cells were grown in Endothelial Cell Basal Medium-2 lacking folic acid. Cells were cultured in a humidified incubator at 37 °C, 5% of CO2, and 95% of air atmosphere with fresh medium replaced every 2 to 3 days. At 95% confluence, cells were subcultured using 0.25% of trypsin-EDTA. For qPCR and western blot analyses, cells were cultured in rat tail collagen-coated 25cm2 flasks (T25) and collected upon reaching 100% confluence.

### Cell viability assay

The viability of hCEMC/D3 cells in the presence of PQQ (1–50 µM) was assessed using the MTT (3-[4,5-dimethylthiazol-2-yl]-2,5 diphenyl tetrazolium bromide) assay. Following 24 h treatment with PQQ, cells were incubated for 2 h at 37 °C with a 2.5 mg/mL MTT solution in PBS. The formazan content in each well was dissolved in DMSO and quantified at 580 nm using a SpectraMax 384 microplate reader (Molecular Devices, Sunnyvale, CA). Cell viability was assessed by comparing the absorbance of cellular reduced MTT in PQQ-treated cells to that of vehicle (DMSO)-treated cells, in both folate-sufficient (FS) and FD conditions (Supplemental file: Fig. S1).

### Animal studies-mouse model of folate deficiency

To examine the *in vivo* effects of FD on BBB integrity, C57BL6/N male wildtype mice were placed on a FD diet containing 0 mg/kg folic acid (supplemented with 1% succinyl sulfathiazole), or with a diet containing 2 mg/kg folic acid designated as the control FS diet (Envigo, Indianapolis, IN, USA). Mice (3–4 weeks) were placed on a FD diet or a FS diet for 5 weeks to induce both systemic and brain FD (12 animals per diet group). This FD diet is similar to that used in our previous study by Sangha et al. (2023) [43], which confirmed the induction of brain FD applying a microbiological assay using Lactobacillus rhamnosus. Following the end of the 5-week period, mice underwent a 10-day PQQ or saline treatment (6 animals per treatment group), with FS or FD diets maintained throughout this treatment period (saline FS:  n= 6, PQQ FS:  n= 6, saline FD: n= 6, PQQ FD:  n= 6). 24 h following the last PQQ/saline treatment, the NaFl assay was performed to assess BBB permeability while in a separate study capillary isolation was performed for gene expression analysis. All procedures were performed in accordance with the Canadian Council on Animal Care guidelines and approved by the University of Toronto Animal Care Committee.

### Isolation of mouse brain capillaries

Brain capillaries were isolated from C57BL6/N male mice (8–9 weeks old) following the 10- day PQQ/saline treatment according to the protocol described by Chan and Cannon (2017) [52], with few modifications [21]. Briefly, animals were anesthetized by isoflurane inhalation and decapitated once a deep anesthetic surgical plane was achieved. Brains were collected immediately, cortical gray matter was removed and homogenized in ice-cold PBS containing calcium plus magnesium and supplemented with 5 mM glucose and 1 mM sodium pyruvate. 30% Ficoll solution was added to the brain homogenates, and centrifuged at 5,800 g for 20 min at 4 °C. The resulting pellet of capillaries was re-suspended in isolation buffer supplemented with 1% bovine serum albumin (BSA) and filtered through a 300 μm nylon mesh. The filtrate containing the capillaries was passed through a 30 μm pluriStrainer. Capillaries were harvested with 50 mL isolation buffer and centrifuged at 1,600 g for 5 min. The resulting pellet containing the purified capillaries was snap-frozen in liquid nitrogen and kept at − 80 °C until further analysis.

### PQQ treatments

For *in vitro* experiments, 80% confluent hCMEC/D3 monolayer cells grown on T25 flasks were treated with PQQ (1 or 5 µM) or dimethyl sulfoxide (DMSO) vehicle (~ 0.08% DMSO in FS or FD media) for 24 h at 37 °C. Following 24 h treatment, cells were collected and processed for analysis. To confirm that there was no toxicity observed at the selected concentrations, a MTT assay was performed on control FS and FD cells (Supplemental File: Fig. S1). For *in vivo* experiments, male C57BL6/N mice (8–12 weeks old) were administered daily intraperitoneal (i.p.) injections of PQQ (20 mg/kg dissolved in saline) or a saline vehicle for 10 consecutive days. This regimen is similar to the one employed in our previous *in vivo *studies demonstrating PQQ’s anti-inflammatory and antioxidant effects in brain tissues, and its ability to upregulate RFC and PGC-1α expression in hCMEC/D3 cells treated with 5 µM PQQ [21, 43]. As our studies utilized a PQQ dose of 20 mg/kg/day, we monitored body weight over 10 days to assess potential toxicities. No significant differences were observed between PQQ and saline-treated mice or between mice on FD and FS diets (Supplemental file: Fig. S2).

### Gene expression analysis

The mRNA expression of the various genes of interest was quantified using qPCR. Total RNA was isolated from hCMEC/D3 cells and mouse brain capillaries using TRIzol reagent following supplier’s protocol (Invitrogen, Carlsbad, CA, USA). RNA concentration (absorbance at 260 nm) and purity (absorbance ratio 260/280) was assessed using NanoDrop One Spectrophotometer (Thermo Scientific, Waltham, MA, USA). RNA (2 µg) was reverse transcribed to cDNA using a high-capacity reverse transcription cDNA kit according to manufacturer’s instructions. Specific human or mice primers for *SLC19A1/Slc19a1* (RFC; Hs00953344_m1/Mm00446220_m1), *SLC46A1/Slc46a1* (PCFT; Hs00560565_m1/Mm00546630_m1), *NRF1/Nrf1* (NRF-1; Hs00602161_m1/Mm01135606_m1), *PPARGC1A/Ppargc1a* (PGC-1α; Hs00173304_m1/Mm01208835_m1), *TFAM* (Tfam; Hs01082775_m1), *TFB1M* (TFB1M; Hs01084404_m1), *TFB2M* (TFB2M; Hs00925025_m1), *IL6/Il6* (IL6; Hs00174131_m1/Mm00446190_m1), *CXCL8* (IL8; Hs00174103_m1), *CCL2* (CCL2; Hs00234140_m1), *CXCL10/Cxcl10* (CXCL10; Hs00171042_m1/ Mm00445235_m1), *PECAM1/Pecam1* (PECAM-1; Hs01065279_m1/ Mm01242576_m1), *CDH5* (VE-cadherin; Hs00901465_m1), *ICAM1* (ICAM1; Hs00164932_m1), *VCAM1* (VCAM1; Hs01003372_m1), *NOS2* (iNOS; Hs01075529_m1), *NOX5* (NAPDH oxidase 5; Hs00225846_m1), *NOS3* (eNOS; Hs01574665_m1), *TJP1/Tjp1* (ZO-1; Hs01551861_m1/Mm01320638_m1), *OCLN/Ocln* (OCLN; Hs00170162_m1/Mm00500912_m1), *CLDN5/Cldn5* (CLDN5;Hs0053349_s1/ Mm00727012_s1), *ABCB1* (P-gp; Hs00184500_m1), *ABCG2* (BCRP; Hs01053790_m1), *SLC2A1* (Glut-1; Hs00892681_m1), *ABCC1* (MRP-1; Hs01561483_m1), *ABCC2/Abcc2* (MRP-2, Hs00960489_m1/Mm00496899_m1), and *SLC22A8/Slc22a8* (Hs01056646_m1/Mm00459534_m1) were obtained from Life Technologies (Carlsbad, CA, USA) for use with TaqMan qPCR chemistry. All assays were performed in triplicates with the housekeeping gene for human/mouse cyclophilin B (*PPIB/Ppib*; Hs00168719_m1/Mm00478295_m1) as an internal control. For each gene of interest, the critical threshold cycle (CT) was normalized to cyclophilin B using the comparative CT method. The difference in CT values (ΔCT) between the target gene and cyclophilin B was normalized to the corresponding ΔCT of the vehicle control (ΔΔCT) and expressed as fold expression (2^−ΔΔCT^) to assess relative difference in mRNA expression for each gene.

### Western blot analysis

Western blot analysis was performed applying our established laboratory protocols [22, 53]. Briefly, hCMEC/D3 cell lysates were prepared using a modified RIPA buffer containing 50 mM Tris (pH 7.5), 150 mM NaCl, 1 mM EGTA, 1 mM sodium o-vanadate, 0.25% sodium deoxycholate, 0.1% sodium dodecyl sulfate (SDS), 1% NP-40, 200 µM PMSF, and 0.1% protease inhibitor. Protein concentrations were measured using the Bradford assay (Bio-Rad, 5000201, Mississauga, ON, Canada) with BSA as the standard. For each sample/lane, 50 µg of total protein was mixed with 1X Laemmli buffer containing 10% β-mercaptoethanol, resolved on a 10% SDS-polyacrylamide gel, and transferred onto a polyvinylidene fluoride membrane overnight at 4 °C. Membranes were blocked for 1 h at room temperature in 5% skim milk in Tris-buffered saline with 0.1% Tween 20, followed by overnight incubation at 4 °C with the following primary antibodies: rabbit polyclonal anti-SLC19A1 (RFC; 1:250, 2–4 µg/mL), anti-SLC46A1 (PCFT; 1:250, 2–4 µg/mL), anti-OCLN (1:250, 1 µg/mL), anti-CLDN5 (1:250, 1 µg/mL), anti-TJP1 (1:500, 0.44 µg/mL), and mouse monoclonal anti-β-actin (1:1000, 0.2 µg/mL). Membranes were then incubated for 1.5 h at room temperature with horseradish peroxidase-conjugated anti-rabbit or anti-mouse secondary antibodies (1:10,000, 0.08 µg/mL). Bands were visualized using enhanced chemiluminescence SuperSignal West Pico Substrate (Thermo Scientific, Waltham, MA, USA) and captured using the ChemiDoc MP Imaging System (Bio-Rad). Protein bands were quantified relative to β-actin using densitometric analysis.

### Enzyme-linked immunosorbent assay (ELISA) analysis

Following treatment with PQQ (1, 5 µM) or DMSO vehicle, hCMEC/D3 culture supernatants were collected, centrifuged at 1500 rpm at 4 °C for 10 min and stored at − 80 °C until the day of analysis. The levels of Il6, Il8, CCL2, CXCL10 in the culture supernatant were quantified using LEGEND MAX Human ELISA Kits following manufacturer’s instructions. Each kit utilized 96-well strip plate pre-coated with Il6, Il8, CCL2 or CXCL10 antibodies, and absorbance was read at 450 nm using a SpectraMax 384 microplate reader (Molecular Devices, Sunnyvale, CA).

### Sodium fluorescein (NaFl) BBB permeability assay

The NaFl assay was performed following published protocols to assess BBB permeability by measuring the diffusion of a NaFl solution from plasma into brain parenchyma [54, 55]. Briefly, NaFl powder (Sigma-Aldrich, F6377-100G) was diluted in 0.9% saline to a final concentration of 30 mg/mL. Each mouse received 100 µL (120 mg/kg, i.p.) of the NaFl and was anesthetized 20 min post-injection. Blood samples (500 µL) were collected from the right ventricle before intracardiac perfusion. Brains were harvested, homogenized in 2 mL of PBS, and mixed with 2 mL of 60% trichloroacetic acid (Sigma-Aldrich) to precipitate proteins. The homogenates were kept at 4 °C for 30 min and centrifuged at 18,000 g at 4 °C for 10 min. Fluorescence was measured using a spectrophotometer at an excitation wavelength of 440 nm and an emission wavelength of 525 nm. The cerebral extraction ratio (CER) was calculated as ([tissue fluorescence]/[g brain])/([serum fluorescence]/[mL blood]) × 100 = CER%.

### Statistical analysis

For all *in vitro *work, experiments were repeated at least three times using hCMEC/D3 cells pertaining to different passages. The *in vivo* experiments with isolated mouse brain capillaries were repeated separately two times. In each individual experiment, the brain capillaries were pooled from 6 animals per treatment group (total *n* = 12/group). For the *in vivo* experiment assessing BBB permeability using the sodium fluorescein assay, samples were collected from 6 animals per treatment group. Results are presented as mean ± SEM. Multiple group comparisons were performed using two-way analysis of variance (ANOVA) with Bonferroni’s post hoc test, with *P* < 0.05 being considered statistically significant. All statistical analysis was performed using Prism 9 software (GraphPad Software Inc., San Diego, CA, United States).

## Results

### Effects of FD and PQQ treatment on folate transporters expression at the BBB

**Fig. 1:**
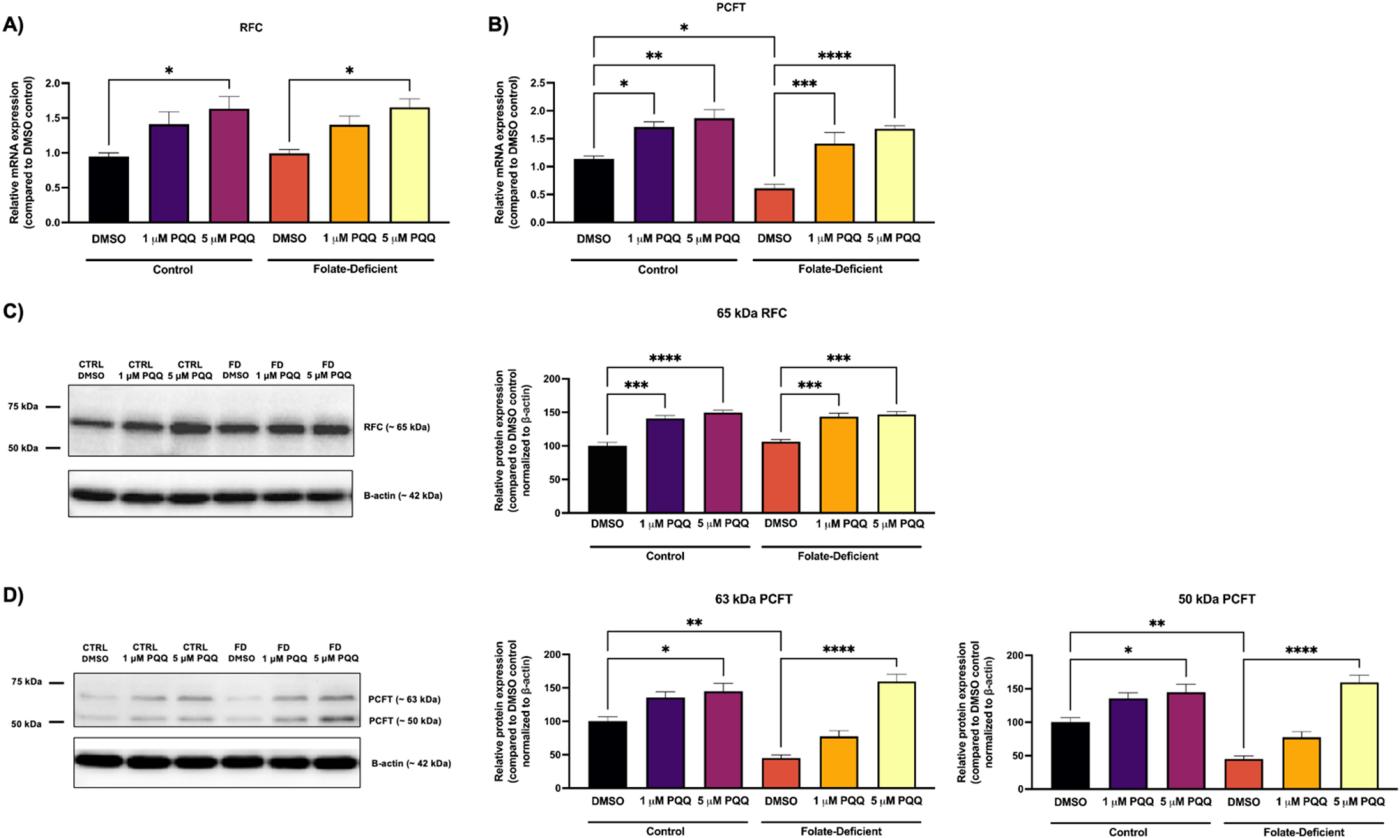
Effects of FD and PQQ treatment on the mRNA expression of folate transporters *SLC19A1* (RFC) and *SLC46A1* (PCFT) in hCMEC/D3 cells. Cells cultured in a FD or FS control medium were treated with PQQ (1 μM or 5 µM) or vehicle (DMSO) for 24 h. (**A**) *SLC19A1* (RFC) and (**B**) *SLC46A1* (PCFT) mRNA levels were measured using qPCR. Cyclophilin B was used as the housekeeping gene. Results are presented as mean relative mRNA expression normalized to the DMSO vehicle control ± SEM from *n* = 4–5 independent experiments using cells from different passages. (**C**) RFC and (**D**) PCFT protein bands were detected by western blot analysis using anti-SLC19A1 and anti-SLC46A1 antibodies. For PCFT, multiple bands were observed, corresponding to different glycosylated forms. β-actin was used as the loading control. Densitometric analysis was performed using Image Lab software (Bio-Rad) to quantify protein band intensities. Protein expression data are presented as mean relative protein expression normalized to the corresponding DMSO vehicle control ± SEM from *n* = 3 independent experiments using cells from different passages. Statistical analysis was performed using two-way ANOVA with Bonferroni’s post-hoc test. Asterisks represent significant differences. *=*p* < 0.05, **=*p* < 0.01, ***=*p* < 0.001, ****=*p* < 0.0001

Using an *in vitro *human BBB model, the hCMEC/D3 cells, we assessed the expression of folate transporters in control FS and FD conditions, with or without PQQ treatment, to investigate the effects of FD and NRF-1 involvement in their regulation. RFC gene expression remained unchanged under FD conditions compared to FS controls (Fig. 1A). However, PQQ (5 µM) treatment for 24 h significantly increased RFC expression by ~ 1.5-fold in both FS and FD conditions (Fig. 1A). In contrast, PCFT gene expression was significantly reduced by ~ 2-fold in FD cells (Fig. 1B). PQQ treatment increased PCFT expression by ~ 1.8-fold in FS cells and by ~ 1.6-fold in FD cells at 1 µM and 5 µM, respectively (Fig. 1B).

Western blot analysis supported the gene expression results, showing no change in RFC protein levels under FD conditions (Fig. 1C), while PCFT protein expression was significantly decreased by ~ 2-fold compared to FS cells (Fig. 1D). PQQ (1 and 5 µM) treatment significantly increased RFC protein expression by ~ 1.5-fold in both FS and FD conditions, relative to vehicle-treated cells (Fig. 1C). PCFT protein expression was increased by ~ 1.6-fold in FS cells and ~ 2-fold in FD cells following 5 µM PQQ treatment (Fig. 1D).

**Fig. 2:**
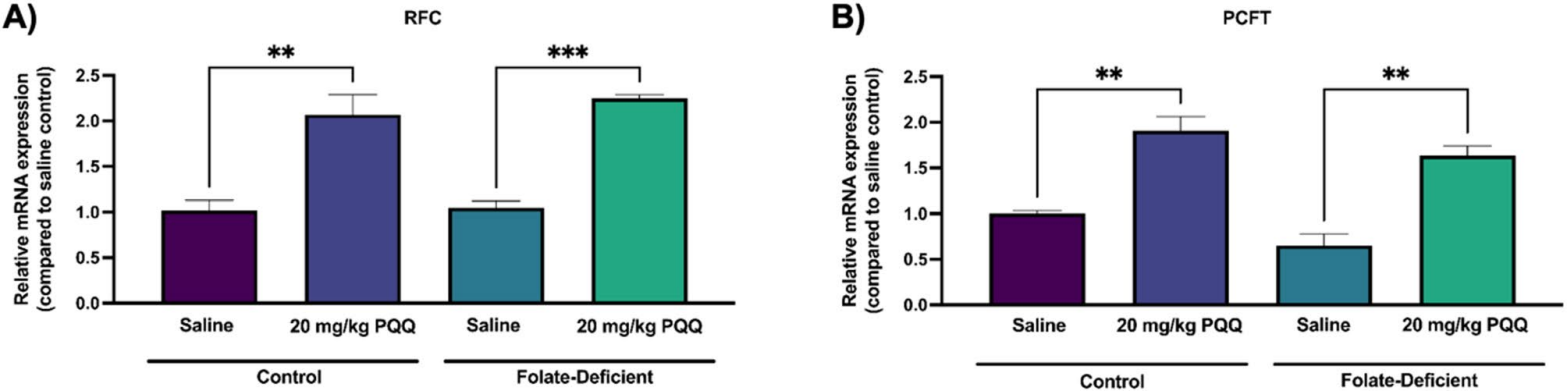
*In vivo* effect of FD and PQQ treatment on the gene expression of the folate transporters *Slc19a1* (RFC) and *Slc46a1* (PCFT) in isolated mouse brain capillaries. Wildtype mice were assigned to FS control (2 mg/kg folate) or FD (0 mg/kg folate) diets for 5 weeks prior to 10-day i.p injections of PQQ (20 mg/kg/day) or saline vehicle. Brain capillaries were isolated 24 h following the last PQQ injection and (**A**) *Slc19a1* (RFC) and (**B**) *Slc46a1* (PCFT) mRNA expression was measured using qPCR. Cyclophilin B was used as the housekeeping gene. Results are presented as mean relative mRNA expression normalized to the saline vehicle control ± SEM from two independent experiments, where each experiment contained brain capillaries pooled from 6 animals per group (total *n* = 12, 2 pools of 6 subjects). Statistical analysis was performed using two-way ANOVA with Bonferroni’s post-hoc test. *=*p* < 0.05, **=*p* < 0.01, ***=*p* < 0.001

*In vivo*, in C57BL6/N mice, a 10-day PQQ treatment (20 mg/kg/day, i.p.) following a 5-week FD diet significantly upregulated RFC and PCFT gene expression by ~ 2-fold in isolated brain capillaries compared to saline-treated FS controls (Fig. 2A, B). Interestingly, FD alone did not alter PCFT gene expression *in vivo*, despite its observed downregulation *in vitro* in hCMEC/D3 cells (Fig. 2B).

### Effects of FD and PQQ treatment on NRF-1/PGC-1α signaling pathway at the BBB

**Fig. 3:**
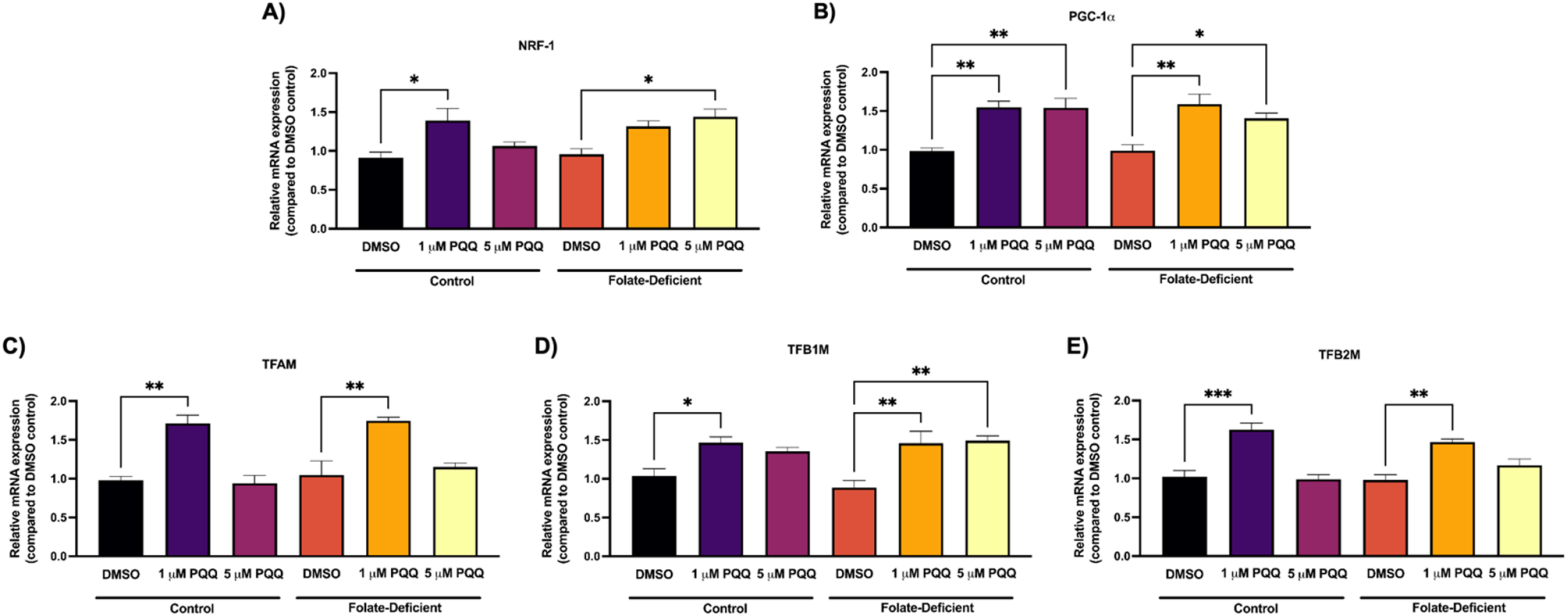
Effects of FD and PQQ treatment on gene expression of PGC-1α/NRF-1 signaling genes and downstream NRF-1 target genes in hCMEC/D3 cells. Cells cultured in a FD or FS control medium were treated with PQQ (1 μM or 5 µM) or vehicle (DMSO) for 24 h. (**A**) *NRF-1*, (**B**) *PPARGC1α* (PGC-1α) (**C**) *TFAM*, (**D**) *TFB1M* and (**E**) *TFB2M* mRNA levels were measured using qPCR. Cyclophilin B was used as the housekeeping gene. Results are presented as mean relative mRNA expression normalized to the DMSO vehicle control ± SEM from *n* = 4–5 independent experiments using cells from different passages. Statistical analysis was performed using two-way ANOVA with Bonferroni’s post-hoc test. *=*p* < 0.05, **=*p* < 0.01, ***=*p* < 0.001

To evaluate the effect of FD and PQQ treatment on mitochondrial function at the BBB, changes in gene expression of the PGC-1α/NRF-1 signaling pathway were assessed. PQQ treatment significantly increased NRF-1 gene expression by ~ 1.5-fold in both FS and FD cells compared to DMSO controls following 1 µM and 5 µM PQQ treatment, respectively (Fig. 3A). Similarly, PGC-1α gene expression was significantly upregulated (~ 1.5-fold) by PQQ (1 and 5 µM) in FS and FD conditions (Fig. 3B). Further analysis of the PGC-1α/NRF-1 signaling pathway in FS and FD cells following PQQ treatment revealed a significant increase in the expression of NRF-1 downstream target genes, mitochondrial transcription factor A (Tfam) by ~ 1.8-fold and B (TFB1M, TFB2M) by ~ 1.5-fold (Figs. 3C-E).

**Fig. 4:**
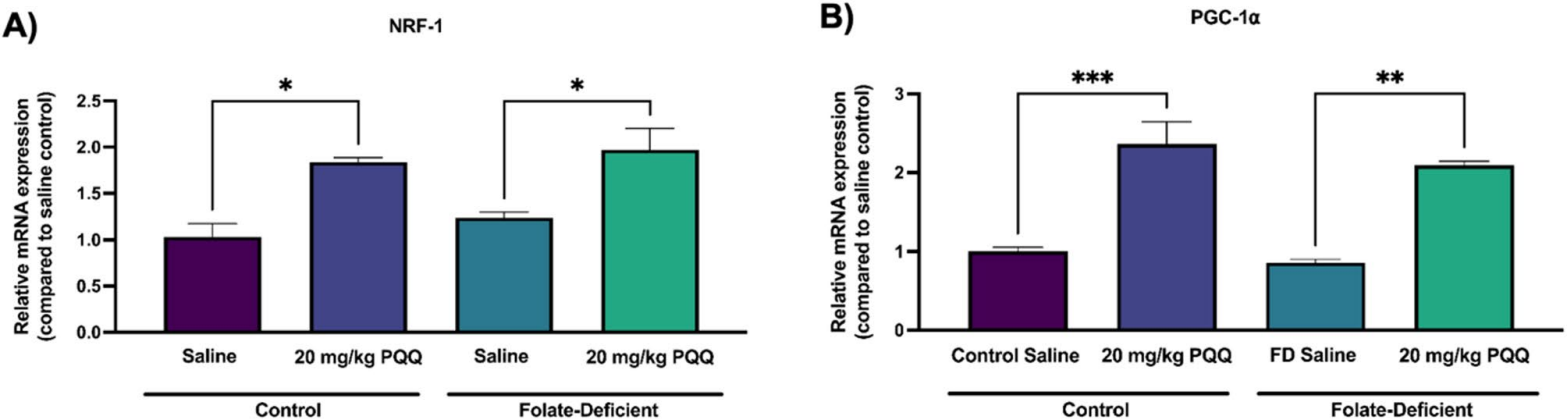
Effect of FD and PQQ treatment on NRF-1 and PGC-1α and gene expression in isolated mouse brain capillaries. Wildtype mice were assigned to FS control (2 mg/kg folate) or FD (0 mg/kg folate) diets for 5 weeks prior to 10-day i.p injections of PQQ (20 mg/kg/day) or saline vehicle. Brain capillaries were isolated 24 h following the last PQQ injection: (**A**) *Nrf-1* and (**B**) *Ppargc1α* (PGC-1α) mRNA levels were measured using qPCR. Cyclophilin B was used as the housekeeping gene. Results are presented as mean relative mRNA expression normalized to the saline vehicle control ± SEM from two independent experiments, where each experiment contained brain capillaries pooled from 6 animals per group (total *n* = 12, 2 pools of 6 subjects). Statistical analysis was performed using two-way ANOVA with Bonferroni’s post-hoc test. *=*p* < 0.05, **=*p* < 0.01, ***=*p* < 0.001

Activation of the NRF-1/PGC-1α pathway by PQQ was further confirmed *in vivo *in isolated C57BL/6 N mice brain capillaries. In both FS and FD conditions, NRF-1 gene expression was significantly increased (~ 2-fold) following 24 h PQQ treatment (Fig. 4A). PGC-1α gene expression was also significantly upregulated following PQQ treatment, with a ~ 2.5-fold increase in FS and a ~ 2-fold increase in FD conditions (Fig. 4B). We could not analyze the expression of NRF-1 downstream targets due to limited RNA yield from mouse brain capillaries.

### Effects of FD and PQQ treatment on inflammatory and oxidative stress markers at the BBB

To investigate the role of FD in inducing inflammatory responses at the BBB *in vitro*, changes in the gene and protein expression of several proinflammatory cytokines and chemokines were assessed in FD hCMEC/D3 cells applying qPCR and ELISA analyses, respectively. We also examined whether PQQ treatment could reverse these effects.

**Fig. 5:**
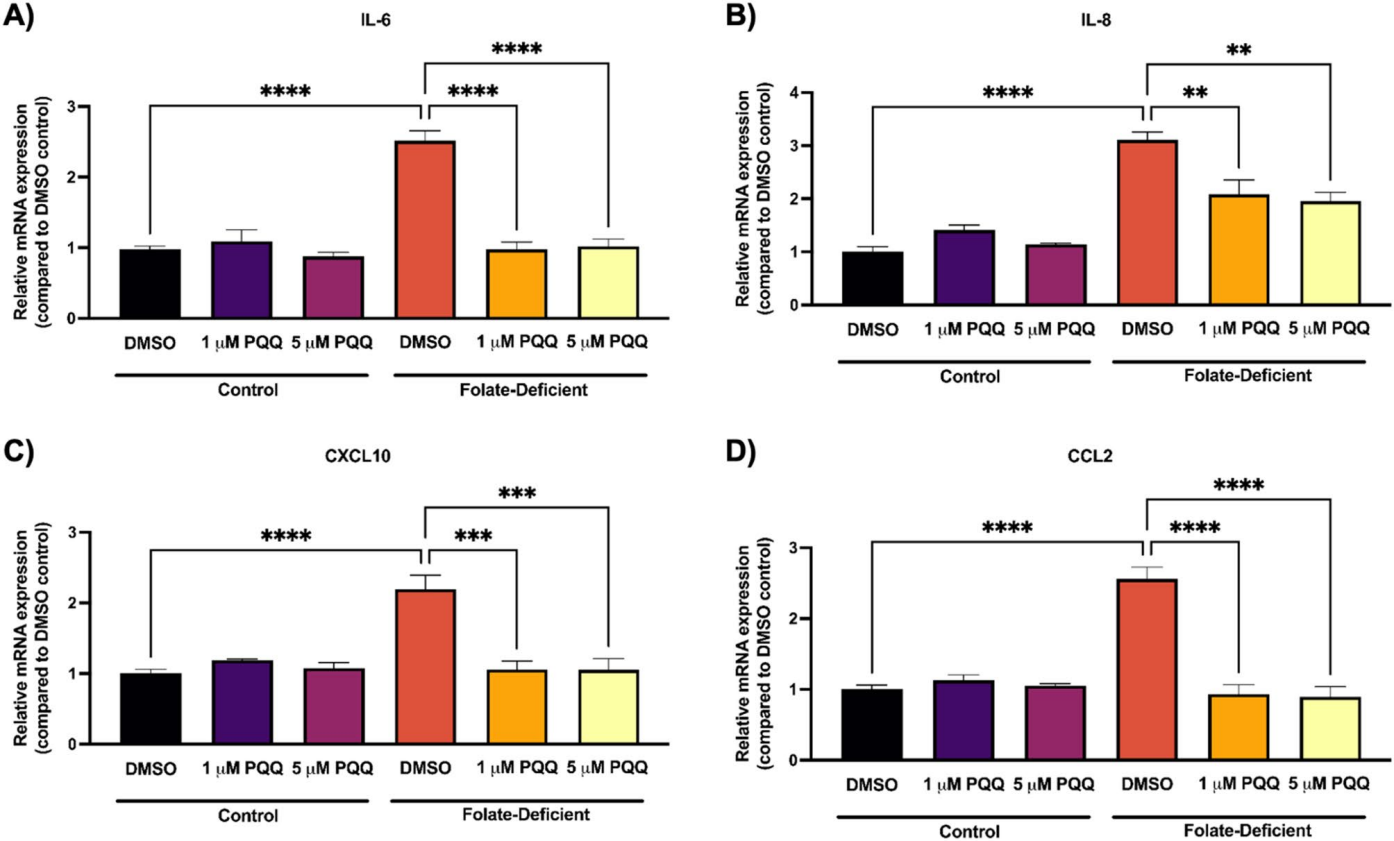
Effect of FD and PQQ treatment on pro-inflammatory cytokines and chemokines gene expression in hCMEC/D3 cells. Cells cultured in a FD or FS control medium were treated with PQQ (1 μM or 5 µM) or vehicle (DMSO) for 24 h. (**A**) *IL-6*, (**B**) *IL-8*, (**C**) *CCL2* and, (**D**) *CXCL10* mRNA levels were measured using qPCR. Cyclophilin B was used as the housekeeping gene. Results are presented as mean relative mRNA expression normalized to the DMSO vehicle control ± SEM from *n* = 4–5 independent experiments using cells from different passages. Statistical analysis was performed using two-way ANOVA with Bonferroni’s post-hoc test. *=*p* < 0.05, **=*p* < 0.01, ***=*p* < 0.001, ****=*p* < 0.0001

**Fig. 6:**
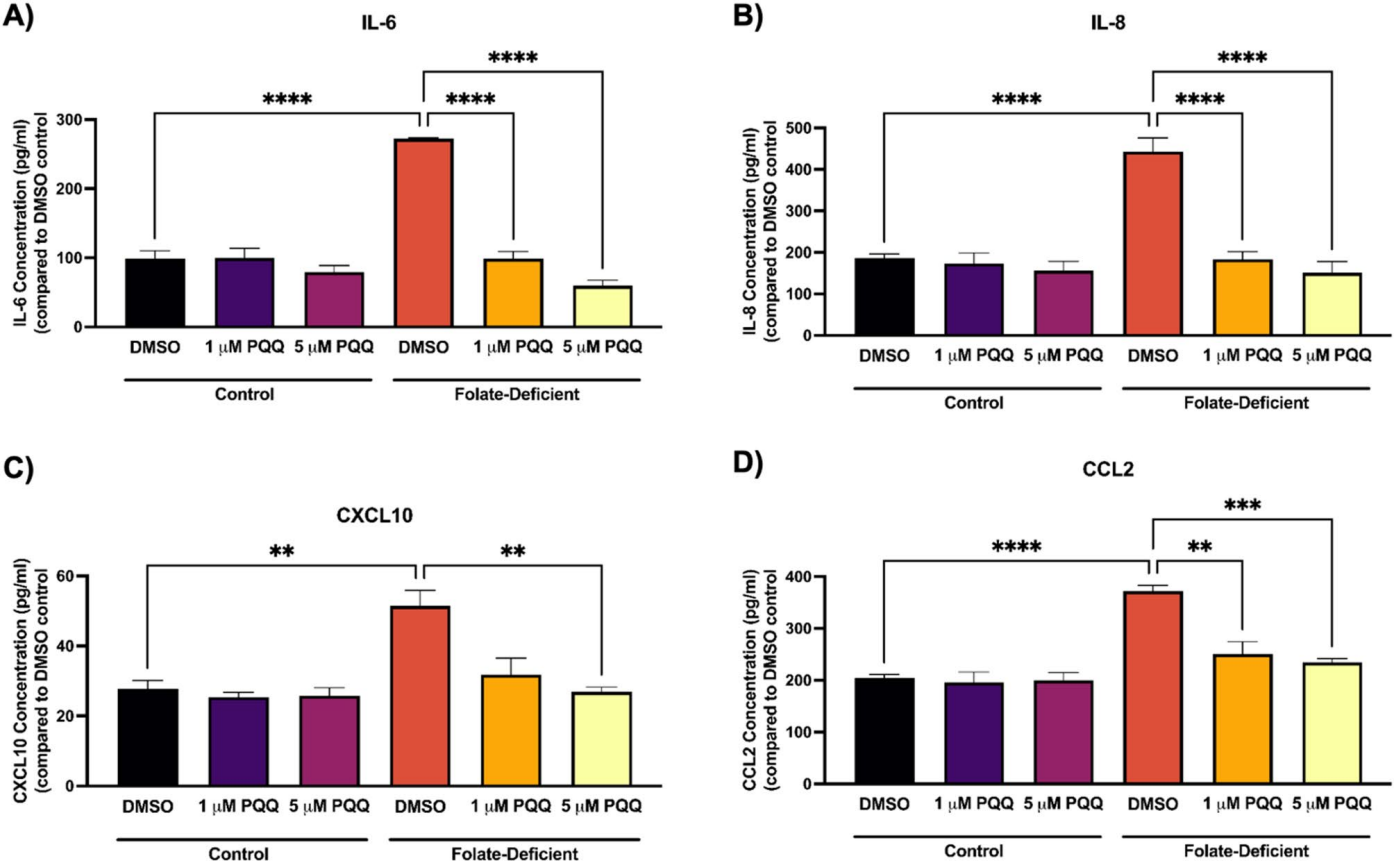
Effect of FD and PQQ treatment on pro-inflammatory cytokines and chemokines protein expression in hCMEC/D3 cells. Cells cultured in a FD or FS control medium were treated with PQQ (1 µM or 5 µM) or vehicle (DMSO) for 24 h. (**A**) IL-6, (**B**) IL-8, (**C**) CCL2, and (**D**) CXCL10 protein levels were measured in the cell supernatant using ELISA. Results are presented as mean relative protein expression normalized to the DMSO vehicle control ± SEM from *n* = 4–5 independent experiments using cells from different passages. Statistical analysis was performed using two-way ANOVA with Bonferroni’s post-hoc test. *=*p* < 0.05, **=*p* < 0.01, ***=*p* < 0.001, ****=*p* < 0.0001

FD led to a significant increase in the gene expression of IL-6 (~ 2.5-fold), IL-8 (~ 3-fold), CXCL10 (~ 2.2-fold), and CCL2 (~ 2.5-fold), while PQQ treatment mitigated these effects by effectively restoring their expression to levels similar to those found in FS conditions (Figs. 5A-D). Consistent with gene expression results, ELISA analysis showed modest but significant increases in IL-6 (~ 2.8-fold), IL-8 (~ 2-fold), CXCL10 (~ 2-fold), and CCL2 (~ 1.8-fold) protein expression under FD conditions, which were reversed following PQQ treatment (Figs. 6A-D).

**Fig. 7:**
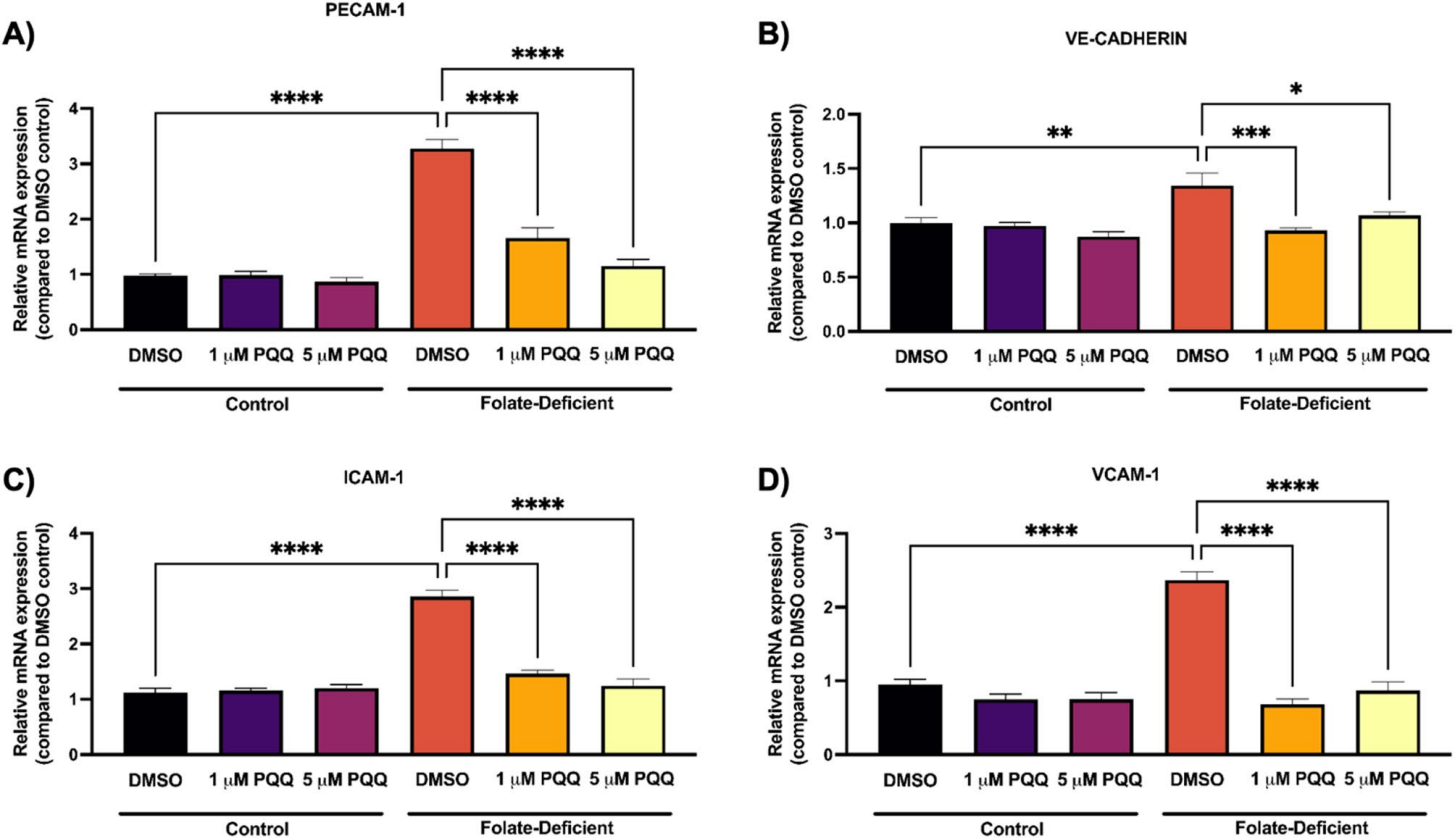
Effect of FD and PQQ treatment on endothelial cells adhesion gene expression in hCMEC/D3 cells. Cells cultured in a FD medium, or FS control medium were treated with PQQ (1 μM or 5 µM) or vehicle (DMSO) for 24 h. (**A**) *PECAM-1*, (**B**) *CDH5* (VE-Cadherin), (**C**) *ICAM-1* and (**D**) *VCAM-1* mRNA levels were measured using qPCR. Cyclophilin B was used as the housekeeping gene. Results are presented as mean relative mRNA expression normalized to the DMSO vehicle control ± SEM from *n* = 4–5 independent experiments using cells from different passages. Statistical analysis was performed using two-way ANOVA with Bonferroni’s post-hoc test. *=*p* < 0.05, **=*p* < 0.01, ***=*p* < 0.001

**Fig. 8:**
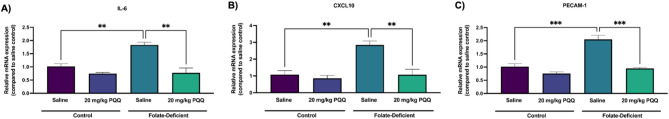
*In vivo* effect of FD and PQQ treatment on the gene expression of pro-inflammatory markers in isolated mouse brain capillaries. Wildtype mice were assigned to FS control (2 mg/kg folate) or FD (0 mg/kg folate) diets for 5 weeks prior to 10-day i.p injections of PQQ (20 mg/kg/day) or saline vehicle. Brain capillaries were isolated 24 h following the last PQQ injection and, (**A**) *Il-6*, (**B**) *Cxcl10* and, (**C**) *Pecam-1* mRNA levels were measured using qPCR. Cyclophilin B was used as the housekeeping gene. Results are presented as mean relative mRNA expression normalized to the saline vehicle control ± SEM from two independent experiments, where each experiment contained brain capillaries pooled from 6 animals per group (total *n* = 12, 2 pools of 6 subjects). Statistical analysis was performed using two-way ANOVA with Bonferroni’s post- hoc test. *=*p* < 0.05, **=*p* < 0.01, ***=*p* < 0.001

To further investigate the effects of FD on inflammation, we examined the gene expression of endothelial cell adhesion molecules, platelet endothelial cell adhesion molecule-1 (PECAM-1), vascular endothelial cadherin (VE-cadherin), intercellular adhesion molecule-1 (ICAM-1), and vascular cell adhesion molecule-1 (VCAM-1) in hCMEC/D3 cells. Here we found that FD led to a significant upregulation of PECAM-1 (~ 3-fold), VE-cadherin (~ 1.4-fold), ICAM-1 (~ 2.8-fold), and VCAM-1 (~ 2.5-fold) genes, which was significantly attenuated by PQQ (1 µM, 5 µM) treatment. (Figs. 7A-D). Furthermore, *in vivo* analysis of isolated brains capillaries from saline-treated FD mice showed significant upregulation of IL-6 (~ 1.8-fold), CXCL10 (~ 2.8 fold), and PECAM-1 (~ 2-fold) gene expression compared to control FS mice, which were also mitigated by PQQ treatment (Figs. 8A-C).

**Fig. 9:**
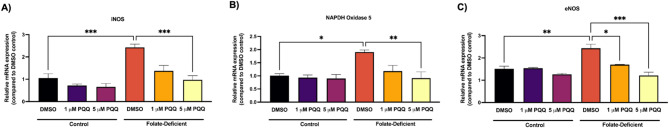
Effect of FD and PQQ treatment on oxidative stress markers gene expression in hCMEC/D3 cells. Cells cultured in a FD or FS control medium were treated with PQQ (1 μM or 5 µM) or vehicle (DMSO) for 24 h. (**A**) *NOS2* (iNOS), (**B**) *NOX5* (NAPDH Oxidase 5), and (**C**) *NOS3* (eNOS) mRNA levels were measured using qPCR. Cyclophilin B was used as the housekeeping gene. Results are presented as mean relative mRNA expression normalized to the DMSO vehicle control ± SEM from *n* = 4–5 independent experiments using cells from different passages. Statistical analysis was performed using two-way ANOVA with Bonferroni’s post-hoc test. *=*p* < 0.05, **=*p* < 0.01, ***=*p* < 0.001

To examine the impact of FD on oxidative stress in hCMEC/D3 cells, we investigated mRNA expression of oxidative stress markers, nitric oxide synthase 2 (NOS2/iNOS), NAPDH oxidase 5 (NOX5), and nitric oxide synthase 3 (NOS3/eNOS). FD cells displayed modest but significant increases in the gene expression of these markers (NOS2: ~2.5-fold, NOX5: ~1.9-fold, NOS3: ~1.4-fold) relative to FS cells. PQQ treatment effectively mitigated these effects, restoring the levels to baseline (Figs. 9A-C).

### Effects of FD and PQQ treatment on BBB integrity

**Fig. 10:**
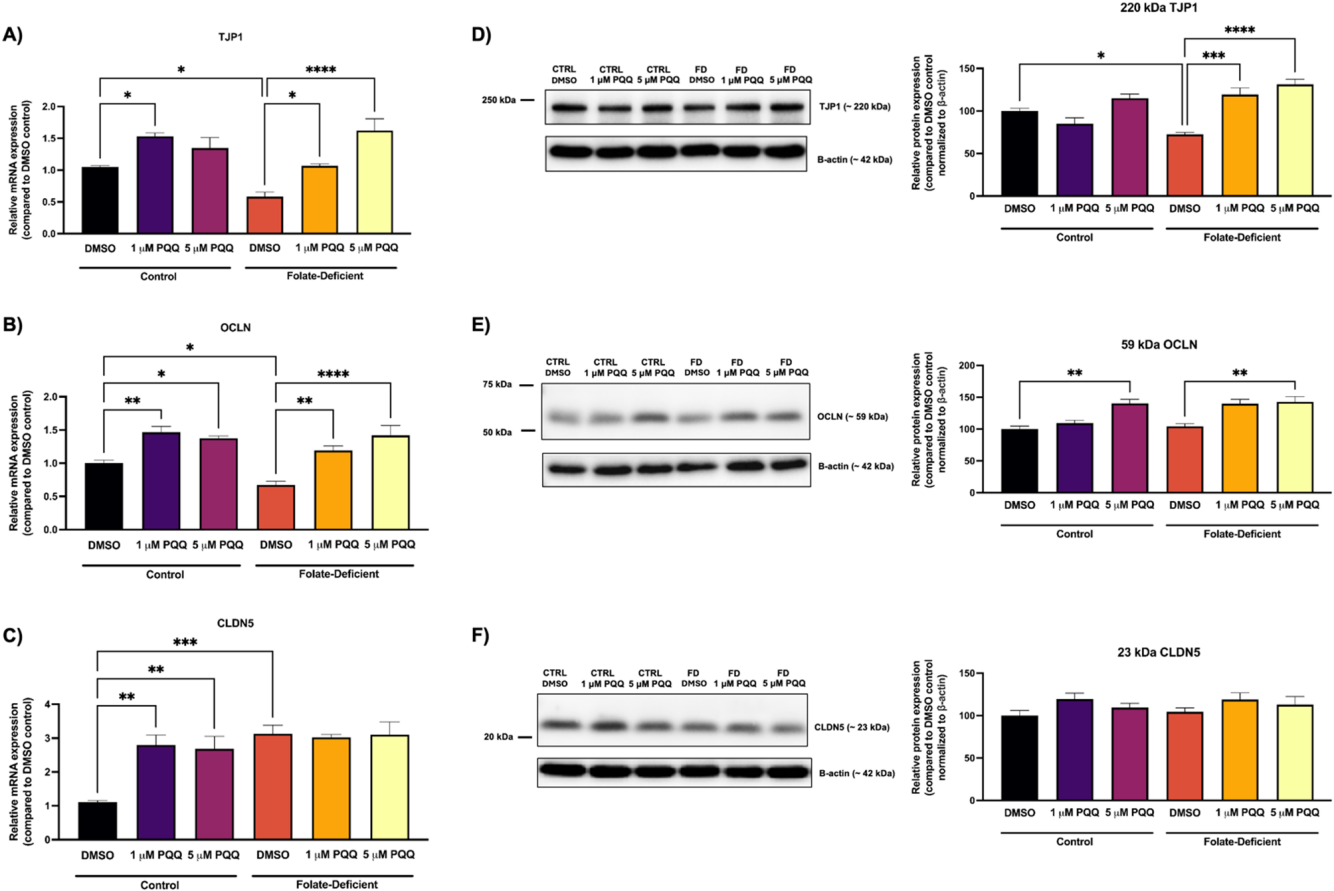
Effects of FD and PQQ treatment on the gene and protein expression of TJ proteins in hCMEC/D3 cells. Cells cultured in a FD or FS control medium were treated with PQQ (1 μM or 5 µM) or vehicle (DMSO) for 24 h. (**A**) Zonula occludens-1 (*ZO-1/TJP1*), (**B**) Occludin (*OCLN*) and, (**C**) Claudin-5 (*CLDN5*) mRNA levels were measured using qPCR. Cyclophilin B was used as the housekeeping gene. Results are presented as mean relative mRNA expression normalized to the DMSO vehicle control ± SEM from *n* = 4–5 independent experiments using cells from different passages. Protein expression of (**D**) TJP1, (**E**) OCLN, and (**F**) CLDN5 was assessed by western blot analysis using anti-ZO-1, anti-OCLN, and anti-CLDN5 antibodies. β-actin was used as the loading control. Densitometric analysis was performed using Image Lab software (Bio-Rad) to quantify protein band intensities. Protein expression data are presented as mean relative protein expression normalized to the DMSO vehicle control ± SEM from *n* = 3-4 independent experiments using cells from different passages. Statistical analysis was performed using two-way ANOVA with Bonferroni’s post-hoc test. *=*p* < 0.05, **=*p* < 0.01, ***=*p* < 0.001, ****=*p* < 0.0001

To further investigate the effects of FD on the BBB, the gene expression of TJ proteins was assessed in hCMEC/D3 cells. These proteins, including zonula occludens-1 (ZO-/TJP1), occludin (OCLN), and claudin-5 (CLDN5), are vital for maintaining the integrity of brain vascular endothelial cells lining the blood vessels. In FD cells, gene expression of TJP1 and OCLN was significantly decreased by ~ 2-fold and by ~ 1.6-fold, respectively, compared to FS cells. PQQ treatment (1 µM, 5 µM) significantly increased the expression of these TJ proteins compared to FS controls (Figs. 10A, B). Interestingly, CLDN5 gene expression was significantly upregulated by ~ 3-fold in FD cells relative to FS cells. PQQ treatment (1 µM, 5 µM) resulted in the significant increase of CLDN5 expression in FS cells by ~ 2.5-fold; however, no differences were observed in FD conditions (Fig. 10C).

Western blot analysis of TJ proteins in hCMEC/D3 cells revealed similar effects for TJP1, with a significant reduction (~ 1.4-fold) in FD cells and a ~ 1.8-fold increase following PQQ treatment in FD cells (1 µM, 5 µM) (Fig. 10D). However, OCLN expression remained unchanged in FD condition, while PQQ treatment (5 µM) increased its expression by ~ 1.5-fold in both FS and FD conditions (Fig. 10E). In contrast, neither FD nor PQQ treatment affected CLDN5 protein levels (Fig. 10F).

**Fig. 11:**
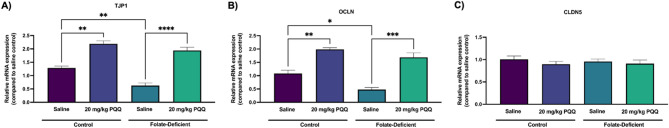
*In vivo* effects of FD and PQQ treatment on the gene expression of TJ proteins in isolated mouse brain capillaries. Wildtype mice were assigned to FS control (2 mg/kg folate) or FD (0 mg/kg folate) diets for 5 weeks prior to 10-day i.p injections of PQQ (20 mg/kg/day) or saline vehicle. Brain capillaries were isolated 24 h following the last PQQ injection and, (**A**) *Tjp1*, (**B**) *Ocln* and, (**C**) *Cldn5* mRNA levels were measured using qPCR. Cyclophilin B was used as the housekeeping gene. Results are presented as mean relative mRNA expression normalized to the saline vehicle control ± SEM from two independent experiments, where each experiment contained brain capillaries pooled from 6 animals per group (total *n* = 12, 2 pools of 6 subjects). Statistical analysis was performed using two-way ANOVA with Bonferroni’s post-hoc test. *=*p* < 0.05, **=*p* < 0.01, ***=*p* < 0.001

Consistent with the *in vitro* findings described previously, *in vivo* analysis of isolated brain capillaries demonstrated a significant decrease in TJP1 (~ 2-fold) and OCLN (~ 2.3-fold) gene expression in FD mice compared to FS mice (Figs. 11A, B). Treatment with PQQ (20 mg/kg/day i.p. for 10 days) significantly increased the expression of both TJ proteins by ~ 4-fold in FD mice and by ~ 2-fold in FS mice (Figs. 11A, B). However, FD and PQQ treatment had no significant effects on CLDN5 gene expression in isolated mouse brain capillaries (Fig. 11C).

**Fig. 12:**
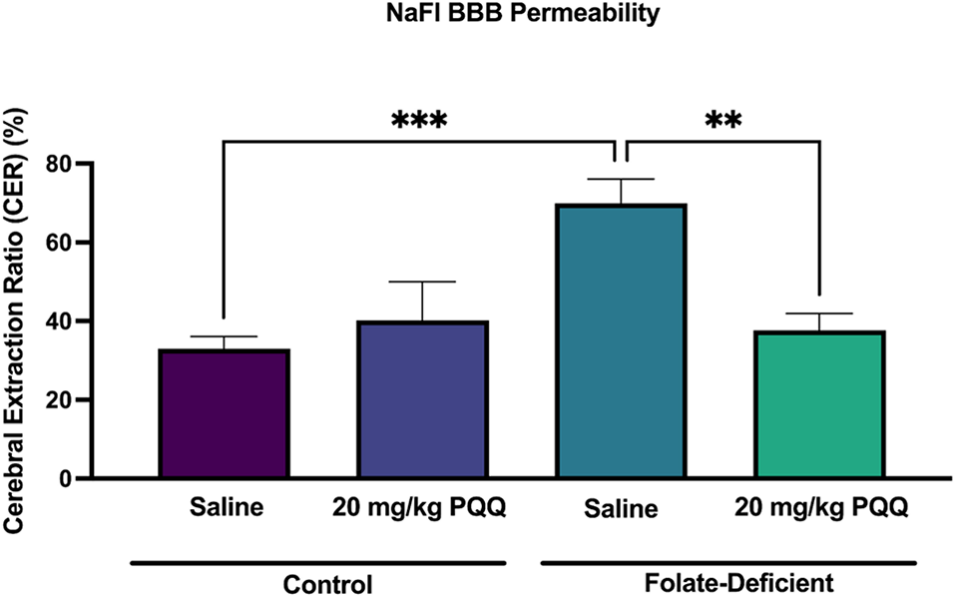
Effects of FD and PQQ treatment on BBB permeability quantified by a sodium fluorescein (NaFl) assay. Wildtype mice were assigned to FS control (2 mg/kg folate) or FD (0 mg/kg folate) diets for 5 weeks prior to 10-day i.p injections of PQQ (20 mg/kg/day) or saline vehicle. 24 h following the last PQQ injection, mice were injected (i.p.) with the NaFl dye. Diffusion of NaFl from plasma into the brain parenchyma was assessed as the indicator of BBB permeability. Results are presented as mean relative cerebral extraction ratio percentage normalized to the saline vehicle control ± SEM, from *n* = 5–6 animals per group. Cerebral extraction ratio (CER) was calculated as ([tissue florescence]/[g brain])/([serum florescence]/[ml blood]) × 100 = CER%. Statistical analysis was performed using two-way ANOVA with Bonferroni’s post-hoc test. *=*p* < 0.05, **=*p* < 0.01, ***=*p* < 0.001

To determine whether the downregulated expression levels of TJ proteins in the FD condition would lead to increased BBB permeability, a sodium fluorescein (NaFl) assay was performed *in vivo*. A significant increase of NaFl penetrance (~ 2-fold) was observed in the brain tissues of FD mice compared with the FS group, suggesting increased brain permeability (Fig. 12). PQQ-treated FD mice showed a significant reduction in NaFl permeability by ~ 2-fold, suggesting that PQQ mitigated the FD-induced disruption of the BBB (Fig. 12).

Finally, no changes in the expression of BBB membrane-associated transporters, including efflux drug transporters BCRP, P-gp, MRP1, MRP2, OAT3 and the glucose uptake transporter GLUT1 were observed in FD hCMEC/D3 cells compared to FS cells and in PQQ-treated cells compared to untreated cells (Supplemental file: Fig. S3, S4). Similarly, no changes in Mrp2 or Oat3 expression were observed in isolated brain capillaries from FD or PQQ-treated mice (Supplemental file: Fig. S4).

## Discussion

The BBB plays a critical role in maintaining brain homeostasis by regulating the transport of essential nutrients, including folates. While the choroid plexus has been considered the main site for brain folate transport, our recent work highlights the BBB as an additional folate delivery route [12, 19–21]. We have shown that PQQ, in addition to its neuroprotective effects, can significantly upregulate RFC expression and transport activity at the BBB, which is relevant in disorders where folate transport through the choroid plexus is compromised [21, 43]. As FD is increasingly associated with physiological abnormalities that may compromise brain function, it is crucial to investigate the direct impact of FD on BBB integrity and whether PQQ can mitigate these effects. In this study, we used a well-established *in vitro* cell culture model of human brain microvessel endothelial cells, the (hCMEC/D3), along with *in vivo *studies, to investigate the impact of FD on BBB dysfunction and assess whether PQQ could mitigate these effects.

Initially, we assessed the effects of FD on the expression of RFC and PCFT at the BBB. Our results revealed that FD significantly downregulated the gene and protein expression of PCFT in hCMEC/D3 cells, while RFC expression remained unchanged (Fig. 1). These results align with our previous findings documenting reduced PCFT expression in primary cultures of mouse mixed glial cells [43]. Interestingly, PCFT expression was unaffected in isolated mouse brain capillaries from mice administered a FD diet compared to those on a control FS diet (Fig. 2), which is consistent with our prior work in mouse brain tissue [43]. Yang et al., also showed that FD altered folate transporter expression, leading to upregulation of RFC and downregulation of PCFT gene expression in mouse hippocampal neuronal cells [56]. In addition, PQQ treatment significantly upregulated PCFT and RFC expression *in vitro* and *in vivo* in both FS and FD conditions (Figs. 1 and 2). These findings build upon our previous studies, which demonstrated RFC upregulation at the BBB by PQQ under FS conditions through PGC-1α/NRF-1 signaling [21] and reveal for the first time that PQQ can also upregulate RFC and PCFT gene expression under FD conditions, providing new insight into how PQQ modulates folate transport at the BBB in conditions associated with CFD. Given that PCFT functions optimally at acidic pH, its contribution to folate transport across the BBB may be limited [13]. In contrast, RFC, located on the luminal membrane of the brain microvessel endothelium, may play a more important role in facilitating folate uptake into the brain parenchyma under physiological pH [22]. Our previous data demonstrated the regulation of RFC by NRF-1/PGC-1α signaling upon activation by PQQ. This was confirmed by chromatin immunoprecipitation (ChIP) and siRNA knockdown studies, establishing that NRF-1/PGC-1α signaling mediates RFC transcriptional regulation [21]. In this study, PQQ increased PGC-1α and NRF-1 expression in both FS and FD conditions (Figs. 3 and 4), resulting in elevated RFC expression in hCMEC/D3 cells and mouse brain capillaries isolated from mice treated with PQQ, further supporting the role of PQQ in modulating folate transporter at the BBB [21]. Moreover, recent work from our group demonstrated that PQQ increased RFC function at the arachnoid barrier (AB), implying that RFC upregulation via NRF-1/PGC-1α at the BBB, the AB, and brain parenchyma may collectively contribute to brain folate uptake in CFD, where folate transport into the CSF is compromised [57].

Neuroinflammation, oxidative stress, and mitochondrial dysfunction have been associated with brain FD and related neurological disorders, and are thought to contribute to neurological dysfunction, ultimately leading to cognitive deficits [41, 58, 59]. However, the impact of these processes on BBB integrity remains unclear. Mitochondrial dysfunction is known to contribute to neurological disorders by compromising the CNS’s ability to meet metabolic demands [41, 42]. Given the role of NRF-1/PGC-1α signaling in mitochondrial function and cellular metabolism, we examined its involvement in BBB regulation under FD conditions. Interestingly, FD did not significantly alter NRF-1/PGC-1α expression in hCMEC/D3 cells and isolated mouse brain capillaries (Figs. 3 and 4). This aligns with our previous study where we found no significant changes in PGC-1α/NRF-1 expression in mixed glial cells and brain tissues of wildtype mice under FD diet [43]. However, FD has been shown to result in increased mitochondrial DNA (mtDNA) deletions and a reduction of overall mtDNA content, potentially disrupting mitochondrial function, impairing energy production, and increasing oxidative stress [39, 43]. Under both FS and FD conditions, our results confirmed that PQQ activates the NRF-1/PGC-1α pathway *in vitro* and *in vivo*, leading to the upregulation of key mitochondrial downstream transcription factors such as TFAM, TFB1M, and TFB2M (Figs. 3 and 4). Interestingly, we observed that 1 µM PQQ induced greater upregulation of mitochondrial genes (e.g., TFAM, TFB2M) than 5 µM, particularly under control conditions (Fig. 3). This nonlinear, gene-specific response is consistent with prior evidence suggesting that PQQ’s biological activity follows a threshold-dependent pattern, where individual genes exhibit optimal activation at distinct concentrations [60–62]. These findings were confirmed across biological replicates and are in line with our previous work showing differential effects of PQQ on PGC-1α, TFAM, and TFB2M expression in mouse arachnoid barrier cells [57]. Further research is needed to clarify the mechanisms underlying these dose-specific effects and to define optimal PQQ concentration ranges for promoting mitochondrial gene expression. Given that mitochondrial biogenesis is a carefully regulated process responding to cellular energy demands, PQQ-driven activation of PGC-1α/NRF-1 may promote mitochondrial activity at the BBB, supporting its integrity and transport efficiency to maintain CNS homeostasis [63, 64].

To further understand the broader effects of FD on BBB function, we investigated its impact on oxidative stress and inflammatory signaling. Sangha et al. reported that FD independently promotes neuroinflammation and oxidative stress in FD mixed glial cells and in mouse brain tissue [43]. In this study, we expanded on these findings by examining alterations in proinflammatory cytokines, chemokines, oxidative stress markers, and adhesion molecules in response to FD and PQQ treatment at the BBB, aiming to understand their dysregulation at this critical interface and potential impact on brain function in CFD-related disorders. We observed a significant upregulation of inflammatory and adhesion molecules both *in vitro*, in FD-treated hCMEC/D3 cells, and *in vivo*, in isolated brain capillaries from mice fed FD diets. PQQ treatment effectively reversed this upregulation, restoring levels to those seen in control mice (Figs. 5, 6, 7 and 8). In addition, a modest elevation in oxidative stress markers was observed in hCMEC/D3 cells in FD conditions, which PQQ treatment mitigated (Fig. 9). These results align with previous studies demonstrating that FD induces inflammation and oxidative stress by promoting microglial activation and proinflammatory cytokine expression in the hippocampus (CA1, CA3, DG) following cerebral ischemia/reperfusion and in oxygen-glucose deprivation (OGD)-treated BV-2 microglia [38]. Mechanistically, FD has been shown to upregulate the Notch1/NF-κB pathway, promoting inflammatory signaling [38, 65]. FD also contributes to oxidative stress by increasing ROS levels and decreasing the expression and activity of key antioxidant enzymes, such as catalase and superoxide dismutase [40]. In addition, FD has also been shown to increase the expression of endothelial cell adhesion molecules, as evidenced by upregulated PECAM-1 expression in an animal model of transient forebrain ischemia [66]. This upregulation is thought to be associated with exacerbated neuronal damage, oxidative stress, and reactive gliosis in the hippocampal CA1 region, highlighting FD’s role in worsening ischemic injury [66]. Notably, increased expression of endothelial adhesion molecules facilitates leukocytes and neutrophil adhesion and trans-endothelial migration, further amplifying inflammatory responses and potentially exacerbating BBB dysfunction [67]. Importantly, in our current study, PQQ treatment effectively reversed these detrimental effects, both *in vitro* and *in vivo*, by reducing inflammatory cytokine expression and oxidative stress markers to near baseline levels. These results support PQQ’s well documented anti-inflammatory and antioxidant properties. In previous studies, PQQ has been shown to suppress pro-inflammatory and pro-oxidant mediators in LPS-treated microglia cells and mouse brains [48]. Similarly, in a D-galactose-induced mouse model, PQQ reduced oxidative stress by decreasing malondialdehyde and ROS levels while upregulating SOD2 expression [68] through modulation of the NF-κB and Nrf2/Keap1 pathways [69].

While inflammation and oxidative stress are well-known contributors to neurological impairments, their impact on the integrity of the BBB is equally critical in several neurological disorders including ASD, Alzheimer’s and Parkinson’s disorders [70–72]. BBB disruptions can allow neurotoxic substances to enter the brain, exacerbating neuroinflammation, metabolic dysfunction, oxidative/nitrosative stress and excitotoxicity [70]. In this study, we examined how FD-induced inflammation and oxidative stress affect BBB integrity, particularly by examining the expression of TJ proteins and BBB permeability. Our findings revealed that FD significantly downregulated the gene and protein expression of key TJ proteins (i.e., TJP and OCLN) in both *in vitro* and *in vivo* BBB models (Figs. 10 and 11). These results align with previous reports demonstrating that FD compromises BBB integrity by disrupting TJs, potentially contributing to the development of FD-associated neurological disorders [73]. Beard et al. showed that elevated homocysteine, a marker of FD [74], decreased the expression of TJ proteins in brain microvascular endothelial cells through modulation of the NMDAR receptor activity [37]. Interestingly, we observed an increase in CLDN5 gene expression in hCMEC/D3 cells maintained in FD condition (Fig. 10C), which contrasts with previous findings showing reduced CLDN5 expression under elevated homocysteine. We hypothesize that CLDN5 increased expression may represent a compensatory response to the FD-induced downregulation of TJP1 and OCLN expression. However, further research is needed to validate this hypothesis. Several studies have shown that early TJ disruption in disease or injury models occurs through downregulation, phosphorylation, or mislocalization of regulatory proteins such as OCLN and TJP1, without immediate changes in CLDN5 [75–77]. For example, in hypoxia and neuroinflammation models, OCLN and TJP1 undergo significant downregulation or redistribution away from tight junctions, while CLDN5 levels remain relatively stable [77–79]. These findings underscore the selective sensitivity of regulatory TJ components to oxidative stress and inflammation. Therefore, the FD-induced downregulation of TJP1 and OCLN observed in our study may reflect early TJ impairment and highlights the importance of assessing both expression and localization in future work. Importantly, we found that PQQ supplementation restored the expression of TJP1 and OCLN under FD conditions (Figs. 10 and 11), consistent with previous reports demonstrating its ability to upregulate tight junction proteins in intestinal porcine enterocyte cells (IPEC-J2) [47]. To assess whether FD-induced downregulation of TJ proteins leads to increased BBB permeability *in vivo*, we performed a NaFl permeability assay. We observed a significant increase in BBB permeability in the brain tissues of FD mice compared to the FS control group (Fig. 12). Treatment with PQQ reversed these effects, restoring permeability levels similar to those observed in control FS mice (Fig. 12). Importantly, serum NaFl fluorescence levels were comparable across all groups, indicating that the observed changes in brain permeability were not driven by differences in systemic tracer availability. In addition, although NaFl is a commonly used marker of BBB integrity, it is also a known substrate of the efflux transporters OAT3 and MRP2 [80]. To address the possibility that PQQ may modulate the expression of these transporters and confound the permeability results, we evaluated OAT3 and MRP2 gene expression in both hCMEC/D3 cells and isolated mouse brain capillaries. No significant changes were observed under FS or FD conditions with PQQ treatment (Supplemental file: Fig. S4), indicating that the increased NaFl accumulation in FD brain tissue, and its reversal by PQQ, are unlikely to result from altered transporter expression. Together, these results confirm that FD disrupts both the structural and functional integrity of the BBB, as evidenced by dysregulated TJ gene and protein expression and increased BBB permeability. PQQ treatment effectively mitigated these effects, suggesting that it may have therapeutic potential for BBB dysfunction in disorders associated with brain FD, even in the absence of folate supplementation.

We acknowledge the limitations of this study. While our *in vitro *studies utilized the well-established immortalized hCMEC/D3 cell line, a representative model of human BBB, it may not fully replicate the complexity of the *in vivo* BBB microenvironment. However, to address this aspect, we conducted *in vivo* studies where we isolated mouse brain capillaries to assess BBB properties, providing a more comprehensive assessment of FD and PQQ effects at this barrier. Additionally, due to the limited yield of mouse brain capillaries, we were unable to assess protein expression levels of folate transporters/receptors, TJ proteins, and pro-inflammatory markers, *in vivo*. Moreover, our *in vivo* work was conducted exclusively in male mice; therefore, we were unable to assess any sex differences in responses to folate deficiency or PQQ treatment. Given that regulatory mechanisms may differ between sexes, future studies will be required in both male and female mice to evaluate potential variability in BBB responses due to sex differences. Despite these limitations, our findings across both models consistently highlight the adverse effects of FD and the neuroprotective properties of PQQ on BBB integrity and function in the context of brain FD.

## Conclusion

In conclusion, this study offers critical insights into the impact of FD on BBB integrity and associated dysfunctions. Our findings demonstrated that FD induced BBB inflammatory responses, oxidative stress, and alteration in TJ proteins expression, collectively leading to BBB disruption. Both *in vitro* and *in vivo* experiments demonstrated the neuroprotective effects of PQQ treatment in FD conditions, effectively mitigating these damaging effects, restoring TJ protein expression and BBB permeability. Additionally, we present novel evidence of FD-induced changes in folate transporter expression in human brain microvascular endothelial cells with PQQ treatment significantly enhancing RFC and PCFT expression in both FS and FD conditions which ultimately could result in increased brain folate delivery in the context of CFD. Given the limitations of current CFD treatment, this work suggests that PQQ may serve as a valuable adjunct therapy, promoting CNS folate uptake and supporting BBB function.

## Electronic supplementary material

Below is the link to the electronic supplementary material.

Supplementary Material 1

## Data Availability

No datasets were generated or analysed during the current study.

## Abbreviations

ASD: Autism spectrum disorder
BBB: Blood–brain barrier
BCSFB: Blood-cerebrospinal fluid barrier
Calcitriol: 1,25-dihydroxycholecalciferol
CFD: Cerebral folate deficiency
CNS: Central nervous system
CSF: Cerebrospinal fluid
CCL2: C-C motif chemokine ligand 2
CXCL10: chemokine (C-X-C motif) ligand 10
CLDN5: Claudin-5
DMSO: Dimethyl sulfoxide
eNOS: Endothelial nitric oxide synthase
FD: Folate-deficient/Folate-deficiency
FRα: Folate receptor alpha
FS: Folate-sufficient
GLUT1: Glucose transporter 1
hCMEC/D3: Human brain microvessel endothelial cells
IL6: Interleukin 6
IL8: Interleukin 8
ICAM-1: intercellular adhesion molecule-1
iNOS: Inducible nitric oxide synthase
MRP: Multidrug resistance protein
mtDNA: Mitochondrial DNA
MTT: 3-(4,5-dimethylthiazol-2-yl)-2,5-diphenyltetrazolium bromide
NaFl: Sodium Fluorescein
NOX5: NADPH oxidase 5
NF-kB: Nuclear factor kappa beta
NRF-1: Nuclear respiratory factor 1
Nrf2: Nuclear factor erythroid 2-related factor 2
OCLN: Occludin
PCFT: Proton-coupled folate transporter
PECAM-1: platelet endothelial cell adhesion molecule-1
PGC-1α: Peroxisome proliferator-activated receptor-γ coactivator-1α
P-gp: P-glycoprotein
PQQ: Pyrroloquinoline quinone
RFC: Reduced folate carrier
RNA: Ribonucleic acid
ROS: Reactive oxygen species
Tfam: Mitochondrial transcription factor A
TFB1M/2M: Mitochondrial transcription factor B
TJ: Tight junction
VCAM-1: Vascular cell adhesion molecule-1
VDR: Vitamin D Receptor
VE-cadherin: vascular endothelial cadherin
ZO-1/TJP1: zonula occludens-1
