# Supplementary material for: Neuroprotective role of pyrroloquinoline quinone in folate deficiency-induced blood-brain barrier disruption
Source: Fluids Barriers CNS. 2025 Jul 22;22:77. doi: 10.1186/s12987-025-00689-y (PMC12285079; doi:10.1186/s12987-025-00689-y)
Supplement: Supplementary file 1 — Supplementary Material 1 [file 12987_2025_689_MOESM1_ESM.docx]

**Supplementary Material**

**Supplemental Figures**


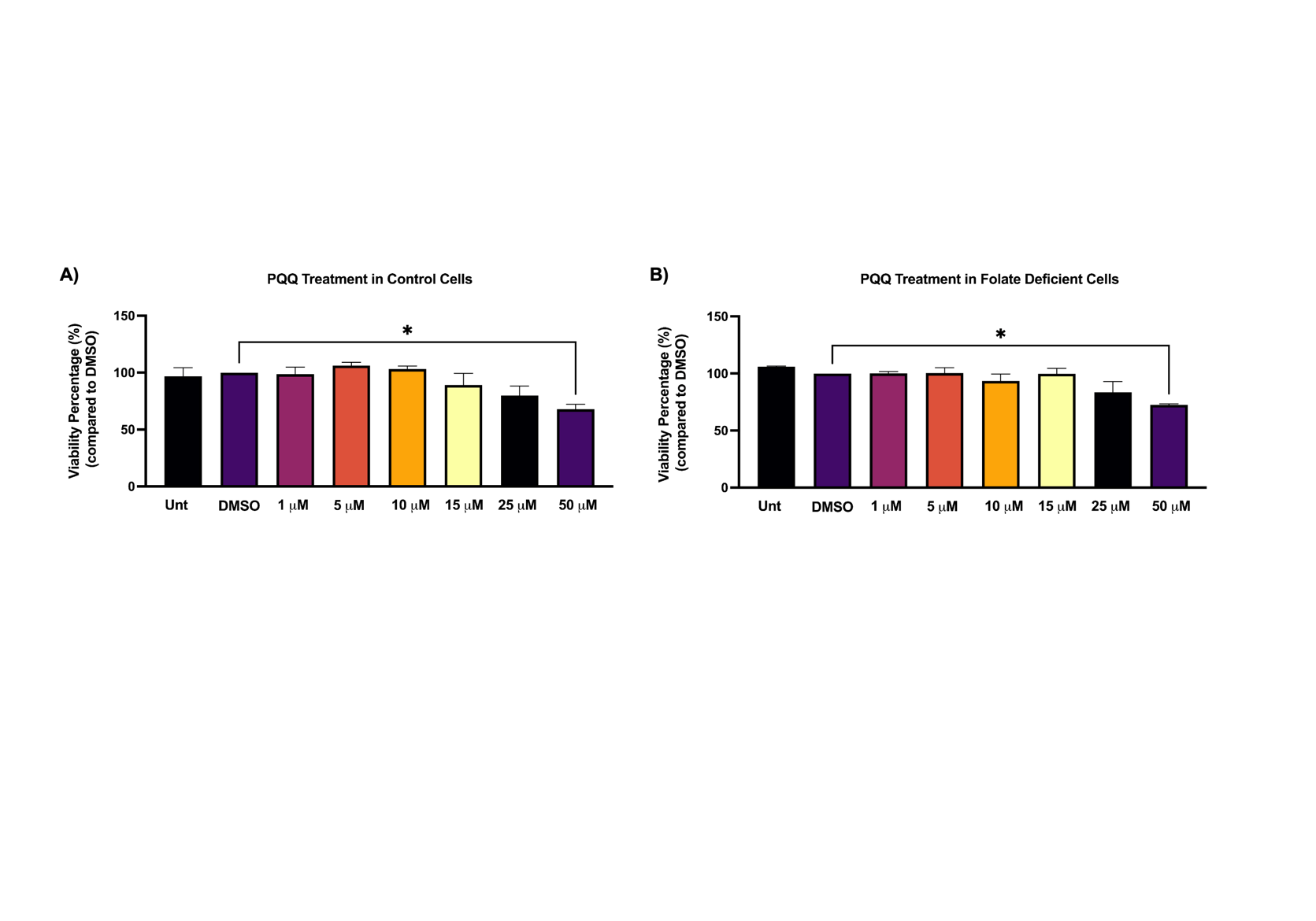


**Figure S1.** Effect of SQQ treatment on FD control and FD hCMEC/D3 cells. MTT assay was conducted to assess the cytotoxic effects of various PQQ doses (1-50 μM for 48h) on hCMEC/D3 cell viability. Exposure to the highest concentration of PQQ (50 μM) significantly reduced cell viability compared to DMSO vehicle-treated cells in the control FS condition (**A**) and in FD condition (**B**). In both control and FD cells, cell viability was unchanged between DMSO vehicle treated and untreated (unt) cells. Results are presented as mean ± S.E.M. for n = 3 independent experiments using cells from different passages. Statistical analysis was performed using two-way ANOVA with Bonferroni’s post-hoc test. **p* <0.05.


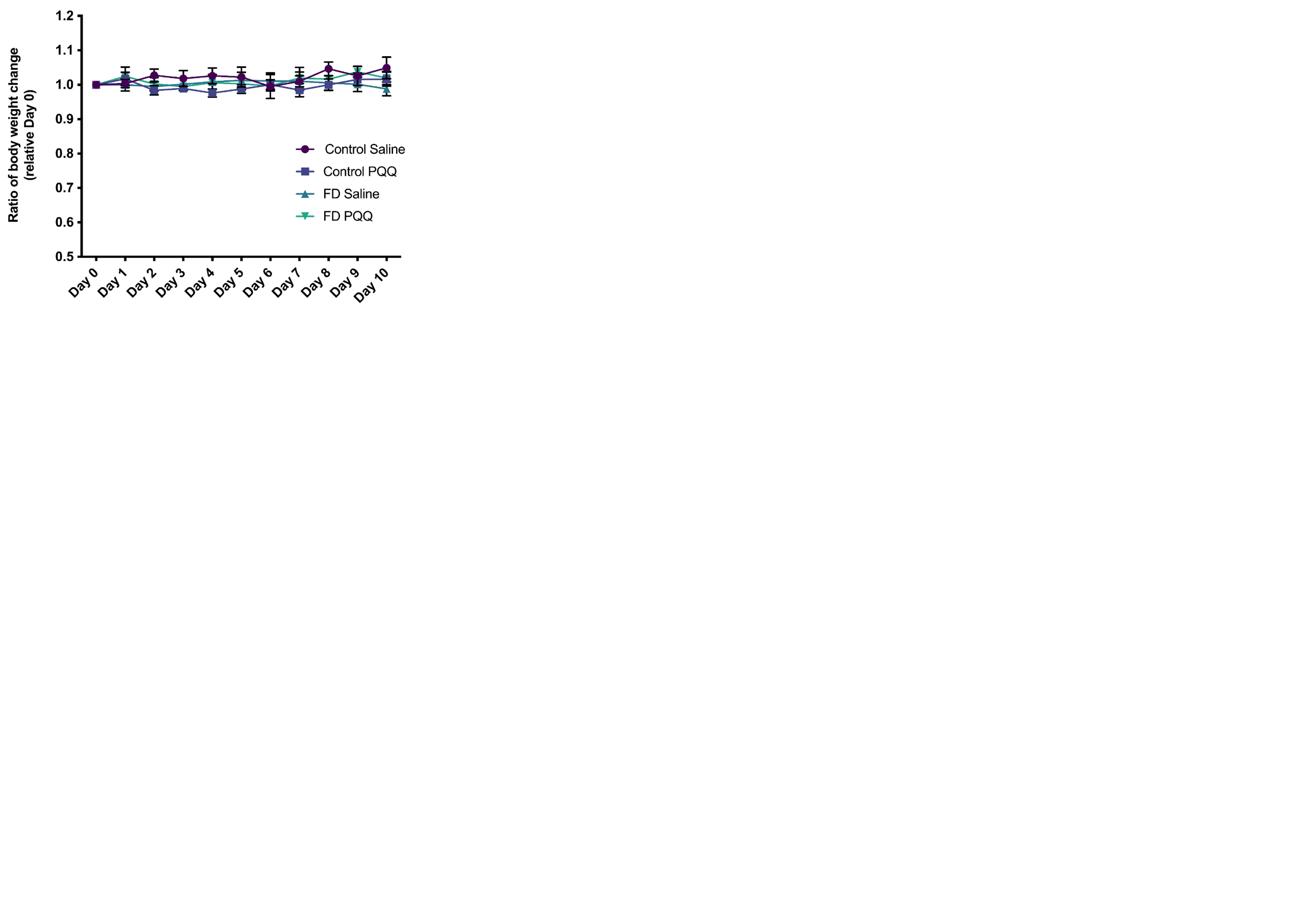


**Figure S2.** Effect of *in vivo* PQQ treatment on the body weight of wildtype mice. Changes in body weight (relative to day 0) was measured over the course of 10-day PQQ (20 mg/kg/day i.p) or saline vehicle treatment period in mice assigned to control FS diet (2 mg/kg folate) or FD (0 mg/kg folate) diet. No significant differences in body weight were observed between the control and FD groups, and PQQ treatment did not alter body weight compared to saline vehicle. Results are presented as a mean change in body weight ± SEM from n = 6 mice/group. Statistical analysis was performed using two-way ANOVA with Bonferroni’s post-hoc test. (*p >0.05)*


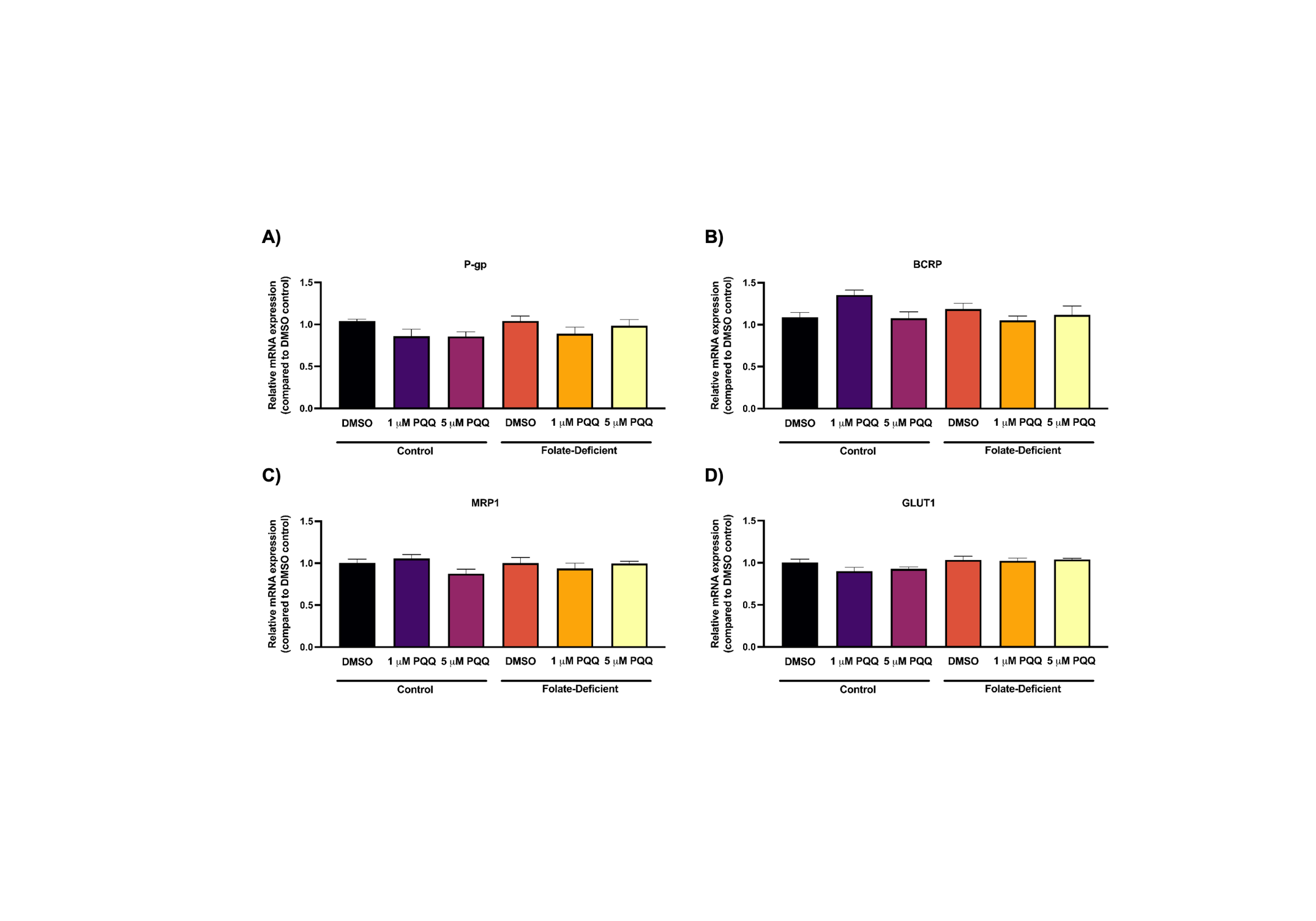


**Figure S3.** Effect of FD and PQQ treatment on BBB transporter genes in hCMEC/D3 cells. Cells cultured in a FD or FS control medium were treated with PQQ (1uM or 5 μM) or vehicle (DMSO) for 24h. **(A)** *ABCB1* (P-gp), **(B)** *ABCG2* (BCRP), **(C)** *ABCC1* (MRP1) and, **(D)** *SLC2A1* (GLUT1) mRNA levels were measured using qPCR. Cyclophilin B was used as the housekeeping gene. Results are presented as mean relative mRNA expression normalized to the DMSO vehicle control ± SEM from n=4-5 independent experiments using cells from different passages. No changes were observed in the expression of BBB transporter genes as determined by two-way ANOVA with Bonferroni’s post-hoc test. (*p<0.05*)

**
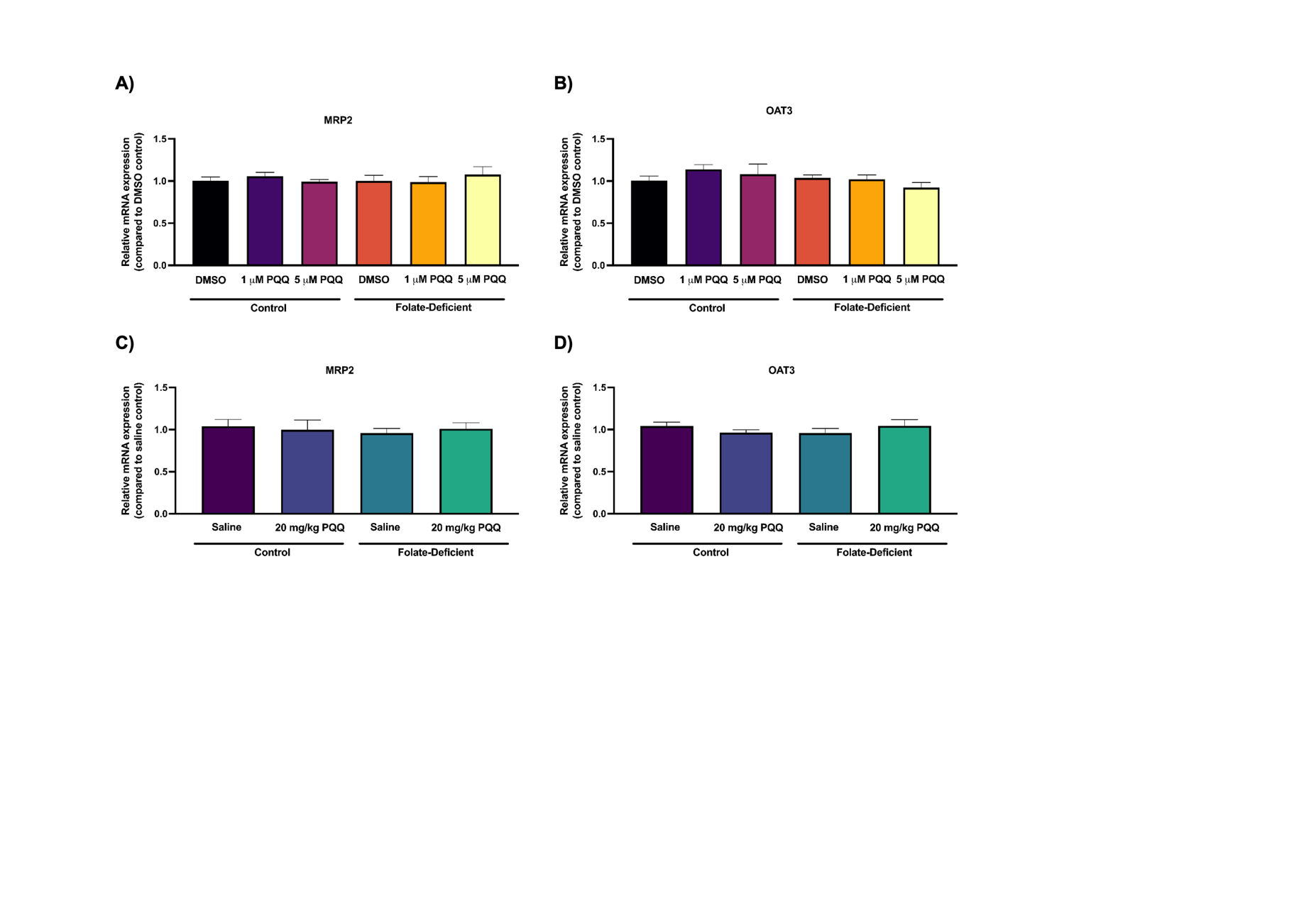
**

**Figure S4.** Effects of FD and PQQ treatment on MRP2 and OAT3 gene expression *in vitro* and *in vivo*. **(A–B)** hCMEC/D3 cells were cultured under FS or FD conditions and treated with PQQ (1 µM or 5 µM) or vehicle (DMSO) for 24 h. **(A)** *ABCC2* (MRP2) and **(B)** *SLC22A8* (OAT3) mRNA expression was assessed by qPCR. Cyclophilin B was used as a housekeeping gene. Data are shown as mean relative mRNA expression normalized to DMSO control ± SEM from n = 5 independent experiments using cells from different passages. **(C–D)** Wild-type mice were fed FS (2 mg/kg folate) or FD (0 mg/kg folate) diets for 5 weeks and treated with daily intraperitoneal injections of PQQ (20 mg/kg/day) or saline for 10 days. Brain capillaries were isolated 24 h after the final injection, and mRNA levels of **(C)** *Abcc2* (Mrp2) and **(D)** *Slc22a8* (Oat3) were measured by qPCR. Cyclophilin B was used as the housekeeping gene. Results represent mean relative mRNA expression normalized to saline control ± SEM from two independent experiments using pooled capillaries from 6 mice per group (total n = 12). Statistical analysis was performed using two-way ANOVA with Bonferroni’s post-hoc test. No significant differences were observed in MRP2 or OAT3 expression in the various treatment conditions.
